# Feedback between stochastic gene networks and population dynamics enables cellular decision-making

**DOI:** 10.1126/sciadv.adl4895

**Published:** 2024-05-24

**Authors:** Paul Piho, Philipp Thomas

**Affiliations:** Department of Mathematics, Imperial College London, London, UK.

## Abstract

Phenotypic selection occurs when genetically identical cells are subject to different reproductive abilities due to cellular noise. Such noise arises from fluctuations in reactions synthesizing proteins and plays a crucial role in how cells make decisions and respond to stress or drugs. We propose a general stochastic agent-based model for growing populations capturing the feedback between gene expression and cell division dynamics. We devise a finite state projection approach to analyze gene expression and division distributions and infer selection from single-cell data in mother machines and lineage trees. We use the theory to quantify selection in multi-stable gene expression networks and elucidate that the trade-off between phenotypic switching and selection enables robust decision-making essential for synthetic circuits and developmental lineage decisions. Using live-cell data, we demonstrate that combining theory and inference provides quantitative insights into bet-hedging–like response to DNA damage and adaptation during antibiotic exposure in *Escherichia coli*.

## Teaser

Agent-based modeling uncovers stochastic mechanisms of stress responses and cell fate decisions.

## INTRODUCTION

Cells make decisions in response to changes in gene expression, which is unexpectedly noisy even among genetically identical cells facing the same environmental conditions (*1*–*3*). Such cellular noise arises from randomness in the biochemical reactions synthesizing proteins. Because these proteins are involved in gene regulatory networks, stochasticity in expression levels can affect cellular functions, cell proliferation, and survival. The interactions between gene networks and population dynamics give rise to phenotypic selection even in clonal populations (*3*–*5*). Phenotypic selection can have functional consequences in development (*6*, *7*), how cells respond to stress (*8*–*10*), and drug resistance (*11*–*13*). Understanding these consequences is important to enhance the function of synthetic circuits inside cells (*14*–*16*).

The Gillespie algorithm is widely used to simulate stochastic gene expression (*1*). The method exactly simulates reaction dynamics at cellular scales, but it implicitly assumes that cells are static and gene expression occurs in isolation from dynamic cellular context. Recent studies have challenged this static view of cells by examining the effect that cell division has on gene expression through mechanisms of partitioning of molecules (*17*–*20*). Some studies showed that when cells compete for growth, the distribution across growing populations differs from isolated lineages as observed in the mother machine (*21*–*23*). However, the differences between such measures are not well understood when gene expression affects cell division.

It is becoming increasingly clear that gene expression noise contributes to cell-to-cell variation in cell growth (*24*, *25*) and cellular timings (*26*). A range of studies have focused on modeling interdivision time through the expression of a division protein hitting a set target level from a fixed basal level (*27*–*31*). The model recovers correlations in interdivision time and cell size compatible with the adder division rule in bacteria. However, a single division protein consistent with time-lapse observations cannot always be identified, and several cues may contribute to phenotypic selection when cells respond to stress or drugs.

More generally, the coupling of gene expression and cell division involves feedback, where gene expression affects cell division frequency, which modulates expression levels. For example, competition between cells decreases the interdivision time and, in turn, increases the frequency with which molecules are partitioned at cell division (*23*). When gene expression affects the interdivision time (*32*, *33*), which we refer here to as division-rate selection, it leads to additional competition. Selection due to differences in division rate is thus distinguished from natural selection arising from a competitive advantage of fast-growing subpopulations.

Natural selection can, in principle, be probed through switching off competition. This can be achieved, for example, by culturing cells in a mother machine (*34*). Yet, differences in division rates are expected to affect expression levels even in isolated cells and can also be engineered artificially (*14*). A quantitative theory of the interactions between gene networks and population dynamics is still missing, and it thus remains elusive how to distinguish natural selection from selection on division rate.

We propose a stochastic framework to model cells as agents that divide in response to intracellular stochastic reaction networks. The model allows us to probe how gene expression contributes to cell proliferation and natural selection and how these effects shape gene expression. Specifically, we show that the division distributions in growing cell populations differ from the first passage distributions of conventional stochastic reaction networks due to the consequences of division rate, natural selection, and cell history. We provide accurate but tractable approximations that provide insights into wide ranges of parameter space and allow us to quantify selection effects from time-lapse observation data.

## RESULTS

### Analytical framework for stochastic agent-based modeling of selection in gene networks

**Fig. 1.**
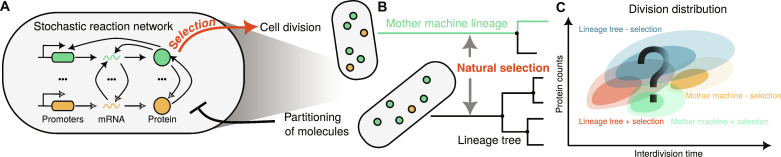
Quantifying interactions between gene networks and population dynamics with agent-based modeling of clonal populations. (**A**) Cartoon of the agent-based model where intracellular reaction network couples to cell division. Intracellular reactions affect cell division rate (red arrow), while partitioning of molecules dilutes cellular expression levels (black repressive arrow). (**B**) Lineage statistics of agent-based models measure distributions across lineage tree resulting from competition of cells through natural selection. A mother machine lineage (green line) follows a single cell in the lineage tree starting from an ancestral cell and following each daughter cell with equal probability. Mother machine sampling avoids natural selection, i.e., competition of cells for growth (red arrow). (**C**) Illustration of division distributions for a cell to divide at a given age and protein count for a lineage tree of cells including division-rate selection and natural selection (red), a lineage tree without division-rate selection (blue), and the corresponding mother machine lineages (green and orange).

We developed a stochastic agent-based formulation of a growing clonal cell population that couples the internal stochastic gene regulatory dynamics and the population dynamics via cell division. In the model, each cell is represented by an agent (Fig. 1A) that contains a gene regulatory network composed of biochemical species *X*_1_, …, *X_N_s__* that react through *R* intracellular reactions. Consequently, the state of each cell is given by its age τ and the molecular content **x**. The cells divide at an **x** and τ-dependent rate γ(**x**, τ) where the dependence of γ(**x**, τ) on **x** encodes the effects of selection on **x**. For example, if γ(**x**, τ) is a monotonically increasing function in **x**, then we have positive selection and negative selection in the case of monotonically decreasing dependence. There is no selection on **x** if the division rate depends only on τ. Such agent-based models of cell populations can be exactly simulated by the extended first division algorithm (see Materials and Methods).

Agent-based stochastic simulations are time- and resource-consuming, especially in the context of parameter inference, when large numbers of parameterizations need to be checked. We can make analytical progress by considering the long-term behavior of the mean number of cells *n*(**x**, τ, *t*) with molecule counts **x** and age τ that grows exponentially with time *t* (section S1). Normalizing this quantity leads to a stable snapshot distribution Π(**x**, τ) = Π(**x**∣τ)Π(τ), where Π(**x**∣τ) is the (conditional) snapshot distribution of molecule numbers for cells with a given age τ, and Π(τ) is the age distribution given by $\Pi \left(\tau \right)\propto \int_{0}^{\tau }\mathrm{ds}e^{\lambda \left(s-\tau \right)}\nu \left(s\right)$ . The age distribution is consistent with the age-structured models of McKendrick and von Foerster (*35*). In their models, the interdivision time distribution ν affecting cell age and division and determining the population growth rate λ is typically assumed to be a fixed parameter or provided by experimental data. However, the (conditional) snapshot distribution is less studied, and, as we will see, interdivision time distribution generally depends on the gene network dynamics in the presence of selection.

We derived the first passage distribution ν(**x**, τ) for a cell to divide at state **x** and age τ, henceforth called the division distribution for brevity

$$\nu \left(x,\tau \right)={\mathrm{me}}^{-\lambda \tau }e^{-\int_{0}^{\tau }\mathrm{ds}\gamma \left(s\right)}\gamma \left(x,\tau \right)\Pi \left(x|\tau \right)$$ (1A)

Here, *m* is the number of offspring at cell division and γ(τ) = *E*_Π_[γ(**x**, τ)∣τ] is the marginal division rate with respect to the conditional distribution Π(**x**∣τ). The snapshot distribution Π(**x**∣τ) of gene expression satisfies a master equation

$$\left[\frac{\partial }{\partial \tau }+\gamma \left(x,\tau \right)-\gamma \left(\tau \right)\right]\Pi \left(x|\tau \right)=Q\left(\tau \right)\Pi \left(x|\tau \right)$$ (1B)

which has to be solved along with a boundary condition that connects the molecule numbers at cell birth and division

$$\Pi \left(x|0\right)=m\int_{0}^{\infty }d\tau \sum_{x^{\prime }\in S}K\left(x|x^{\prime }\right)\nu \left(x^{\prime },\tau \right)$$ (1C)

Biochemical reactions are encoded by the transition matrix

$$Q_{x,x^{\prime }}\left(\tau \right)=\sum_{r=1}^{R}w_{r}\left(x^{\prime },\tau \right)\left(\delta_{x,x^{\prime }+v_{r}}-\delta_{x,x^{\prime }}\right)$$ (2)

where δ is the Kronecker delta, *w_r_*(**x**, τ) are the (potentially cell cycle–dependent) reaction propensities, and *v_r_* is the reaction stoichiometry. The partitioning kernel $K\left(x|x^{\prime }\right)=\frac{1}{2}K_{1}\left(x|x^{\prime }\right)+\frac{1}{2}K_{1}\left(x^{\prime }-x|x^{\prime }\right)$ where *K*_1_(**x**∣**x**′) and *K*_1_(**x**′ − **x**∣**x**′) are the marginal distributions of molecules inherited by the two daughter cells from the mother cell with intracellular state **x**′.

The theory highlights the intricate feedback that exists between gene expression and cell division and underlies division-rate selection. The division rate multiplies the gene expression distribution in the division distribution (Eq. 1A), meaning that cells where γ(**x**, τ) is low are underrepresented, while cells with high division rate are overrepresented. The division distribution then determines the distribution at cell birth (Eq. 1C) and the time evolution of the gene expression distribution (Eq. 1B). The latter differs from chemical master equation models as the frequency of gene expression phenotypes is not only determined by biochemical reactions but modulated by division-rate selection throughout the cell cycle.

Natural selection contributes to this feedback through the exponential dependence of the division distribution on the population growth rate. Cells dividing slower than the population doubling time are underrepresented in the division distribution compared to cells that divide faster. The strength of natural selection is controlled through the number of offspring at cell division. For example, for a lineage tree of cellular agents, we have *m* = 2, and the population growth rate λ needs to be computed self-consistently through normalizing Eq. 1A, while, in the mother machine setting, i.e., considering an isolated cell lineage, we have *m* = 1 and λ = 0. The difference between mother machine lineages and lineage trees provides a measure of natural selection (*36*–*39*). The theory extends to cell growth and cell size control through an effective division rate (see Materials and Methods) and thus provides a coarse-grained view of gene expression–induced selection effects.

### Division-rate versus natural selection in the telegraph gene expression model

**Fig. 2.**
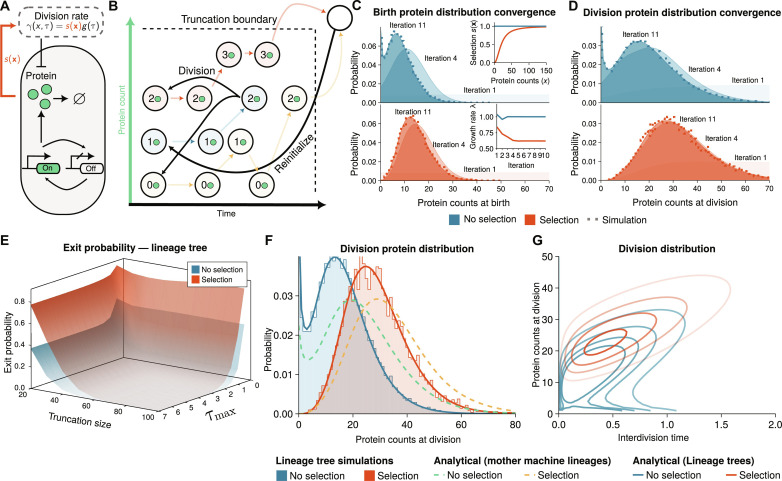
FSP enables accurate prediction of selection on gene expression noise. (**A**) Schematic illustration of the agent-based model. Intracellular dynamics of stochastic gene expression are modeled by the telegraph model for bursty expression of a protein. The protein is binomially partitioned at cell division. The selection effect is introduced through a division rate increasing with expression (details in section S3A). (**B**) Illustration of the finite state projection (FSP) algorithm. Intuitively, gene expression (light arrows) drives cells to either commit to division or cross the truncation boundary, leading to their states being reinitialized (dark arrows). (**C** and **D**) Birth and division distributions for the model converge in a few iterations and agree with agent-based simulation of lineage trees obtained using the first division algorithm (Materials and Methods; see fig. S1 for mother machine lineages). Top inset of (C) shows the used selection functions [*s*(**x**) = 1 for no selection, *s*(**x**) = 1/((20/*x*)^2^ + 1) for selection on the gene expression]. Bottom inset shows the convergence of the computed population growth rate λ (see Materials and Methods). (**E**) The exit probability due to FSP truncation decreases with truncation size and time horizon τ_max_. (**F**) Summary distributions without (blue solid line) and with selection (red solid line) are compared to direct simulations of the lineage trees (shaded areas). Our method is in agreement with the simulation results. Distributions obtained using mother machine lineages (dashed) are also shown. (**G**) FSP solution of the agent-based model predicts bimodal division distribution without division-rate selection (blue lines correspond to levels of equal probability) and an unimodal division distribution in the presence of division-rate selection.

To study selection effects, we consider the telegraph model (Fig. 2A) where a promoter switches between active and inactive states. Once the promoter is activated, a protein is produced in random bursts. Selection in our model is introduced through the division rate, which we assume to be of the form

γ(x,τ)=s(x)g(τ) (3)

where *s*(**x**) describes the effects of gene expression levels **x** on the division rate and *g*(τ) is the division rate in the absence of selection on the gene expression (see also Materials and Methods on modeling growth rate–dependent selection). Our main equations (Eqs. 1A to 1C) cannot be solved in closed form because they are essentially an infinite system of coupled integro–ordinary differential equations (ODEs). An analytical solution is only known in the special case with deterministic divisions and without selection (*20*, *40*, *41*).

To overcome this challenge, we developed a powerful approximation based on the finite state projection (FSP) method (see Fig. 2B and Materials and Methods). In brief, the method consists of restricting the dynamics to a finite subset of the state space X ⊂ *S* for **x** and solving the dynamics of Eqs. 1A to 1C up to a finite cell age τ_max_. This is akin to what is done in the standard FSP method (*42*), but, in addition, we introduce (i) cell division events that split the dividing cell via partitioning of molecules leading to *m* newborn cells and (ii) events that reinitialize single-cell trajectories once they leave the prescribed state space (Fig. 2B).

For the case without selection, we set the expression-dependent selection function in the telegraph model to be *s*(**x**) = 1 and a Hill function for positive selection (Fig. 2C, top inset). We computed the birth and division distributions for a fixed truncation size (Fig. 2, C and D) along with the corresponding growth rate (Fig. 2C, bottom inset). We observe that the iterative scheme converges (Fig. 2, C and D) and the method allows us to compute the exit probability that arises from state space and cell age truncation (Eq. 4 and Fig. 2E). Equation 8 corresponds to an Euler-Lotka equation of population dynamics (*43*) up to an error term that involves the exit probability. The exit probability measures the proportion of cells that reach the truncation boundary and are thus reinitialized instead of dividing. It decreases monotonically with maximum age τ_max_ and truncation size (Fig. 2E) guiding the accuracy of the approximation. The resulting FSP solution (Fig. 2, C and D, shaded area) also agrees well with simulations using the first division algorithm (dots; see Materials and Methods).

Our analysis predicts bimodal distributions without selection on the gene expression, while the distributions with selection are unimodal (Fig. 2, F and G). Bimodality in the absence of selection arises from slow promoter switching (*44*). Such long lived transcriptional states can arise from transcription factor–mediated looping of DNA as observed in the lactose operon of *Escherichia coli* (*45*). To clarify whether the absence of the zero mode is due to the slow-growing subpopulation being outcompeted or due to division-rate selection on the gene expression, we also computed the division distributions of mother machine lineages (Fig. 2F). We observe qualitatively similar division protein distributions for both population lineage trees and mother machine lineages and division-rate with natural selection in the population skewing distributions toward lower expression levels. Intuitively, this can be understood through Eq. 1A, which is proportional to the division rate, while division times longer than the population doubling time are exponentially suppressed. Division-rate selection on the gene expression accounts for the absence of the slow subpopulation in both measures. This highlights that selection in the telegraph model is primarily driven by division rate.

### Growth feedback reinforces lineage decisions in multi-stable gene regulatory networks

**Fig. 3.**
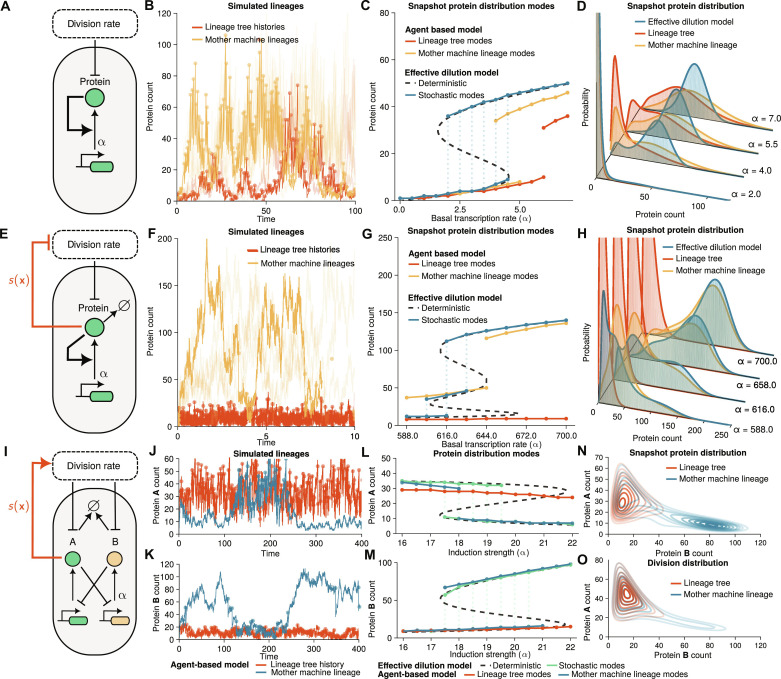
Selection reinforces lineage decisions in cellular switches. (**A** to **D**) Agent-based model of transcriptional feedback. (A) Illustration of the transcriptional feedback model where protein expression promotes its production with parameter α corresponding to a basal transcription rate. Proteins are partitioned binomially at cell division. (B) Agent-based simulations of lineage tree histories and mother machine lineages (α = 4.0) obtained using the first division algorithm (see Materials and Methods) display switching between low– and high–protein expression states. Individual cell divisions are shown as dots to indicate the timescale of cell divisions. (C) Modes (local maxima) of the snapshot protein distributions. FSP solutions of the agent-based model predict bimodal distributions of mother machine (orange) and lineage trees (red) over intermediate basal production rates. The EDM (black and blue) agrees well with the mother machine solution. (D) FSP solutions show a transition from unimodal to bimodal distributions. (**E** to **H**) Agent-based model with transcriptional and growth feedback [*s*(**x**) = *k*_2_/((*x*/*K*_2_)^4^ + 1)] where protein synthesis inhibits division rate and promotes its own production rate. EDM and mother machine lineages have three stable modes, while lineage trees show that fast-dividing cell lineages take over the population. (F) Agent-based simulations of mother machine lineages (basal transcription rate α = 644.0) show switching between low and high protein levels, while, in lineage tree histories, the fast-dividing lineages determine the cell fate. (**I** to **O**) Agent-based model of the genetic toggle switch. Induction strength α corresponds to the maximal transcription rate of protein B. Protein A is under selection via selection function of the form *s*(**x**) = *k*_3_/((*K*_3_/*x*_A_)^2^ + 1). (N to O) Snapshot and division protein distribution display multimodality and a long tail, respectively, in the mother machine lineages (blue, α = 18.9). The fast-dividing subpopulation is selected in the lineage trees (red). Parameters and model details in section S3 (B and C).

We wondered whether simple patterns of selection are important for cell fate decisions. Traditionally, cell differentiation is associated with multi-stability in gene regulatory networks shaping Waddington landscapes (*46*). It is known that protein expression modeled by the birth-death process leads to unimodal distributions both in mother machine and lineage trees (*22*). We therefore considered positive feedback loops that are commonly associated with bistability and bimodal distributions (Fig. 3A). In the model without selection, a protein is expressed from a promoter, which, in turn, increases its own expression. The production rate is modeled as constant basal transcription rate α along with a Hill-type function creating a positive feedback loop. Simulation of population histories [a random lineage of a lineage tree; (*36*)] and a mother cell lineage show large fluctuations (Fig. 3B), leading to long-tailed or bimodal distributions (Fig. 3, C and D).

This phenomenon can be understood in terms of slow switching between discrete states of an effective dilution model (EDM). These effective reaction networks add one dilution reaction per species to the reaction network and can be simulated easily using ODEs or Gillespie simulations. Our analysis of the ODE model of the network reveals the S-shaped multi-stable response to changes in the basal transcription rate (Fig. 3C, dashed black line). Characteristically, traces simulated through the Gillespie simulations switch between two equilibria of high and low gene expression that correspond to modes in the stationary probability distributions (Fig. 3C, solid blue line). Both the lineage tree and mother machine lineages show bimodal snapshot (Fig. 3C) and division distributions (fig. S2) and thus qualitatively agree with the EDM over large parameter regimes. However, a quantitative comparison of the snapshot protein distributions reveals different noise characteristics. EDM gives a better approximation to the mother machine lineage formulation than the lineage trees but underestimates the noise because it provides only an effective description of cell divisions (Fig. 3D).

Because of the presence of random switching, differentiation of this circuit can only be achieved when an external parameter is varied. Yet, how to design circuits that differentiate irreversibly in the absence of fine-tuned signals remains unclear. To this end, we study an extended circuit where additional feedback is introduced to the model via division rate (Fig. 3E). Cell division is modulated through the repressive Hill-type selection function. The extra feedback changes the parameter regime where multimodality appears and in some parameter regimes introduces an additional steady state in the EDM (Fig. 3G).

Simulations of mother machine lineages show switching between the tree expression levels with very slow cell divisions in the highly expressed states (Fig. 3F, orange line). In stark contrast, the histories of population lineage trees display fast divisions selecting only the low-expression state (Fig. 3F, red line) due to the negative feedback between expression and division rate. The higher expression level states in a population get rapidly outcompeted by fast-dividing cells. Comparing the distribution modes of the different measures, we observe that the EDM model explores all three states (Fig. 3, G and H, and fig. S3). The mother machine lineages mostly explore the two high expression states corresponding to slow-dividing cells, while the population lineage trees display exclusively the lowly expressed state that promotes fast cell proliferation. A similar effect, with the fast-dividing subpopulation being selected in lineage trees, is observed when removing the transcriptional feedback loop, which also includes an additional steady state due to growth feedback (fig. S4).

We asked whether selection could provide a general mechanism for lineage decisions beyond the single-gene feedback models. We considered the genetic toggle switch as a common motif in synthetic biology, which comprises two antagonistic proteins inhibiting each other (Fig. 3I). The protein A is assumed to be under selection through the Hill-type selection function. The circuit dynamics are nontrivial as selection only acts on protein A, while the dynamics are tightly coupled with protein B.

To analyze the balance between the division-rate selection and natural selection, we again compared the mother machine lineages with lineage trees. The trajectories of the mother machine lineages explore fast- and slow-dividing states, while lineage tree histories settle into the fast-dividing state where protein A is more highly expressed (Fig. 3, J and K). The effects are confirmed by estimating the modes of the distribution computed using FSP where mother machine lineages are bimodal in close agreement with the EDM (Fig. 3, L and M). In contrast, the protein distributions in lineage trees are unimodal (Fig. 3, L to N). We observe that the bimodality in protein distributions of mother machine lineages was weaker at division (Fig. 3N) than in snapshots (Fig. 3O), implying that division-rate selection shapes the balance between these states.

The effect of division-rate selection in mother machine lineages can be seen as an overrepresentation of slow-dividing cells in snapshots compared to the division distributions that occur because cells spend more time in these slow states. This suggests that division-rate selection acts on division times in mother machine lineages in similar directions to natural selection on lineage trees. However, only because of natural selection, do fast-dividing cells eventually outcompete the slow-dividing ones (figs. S6 and S7). The coupling between gene expression and growth thus represents a robust strategy to implement cell fate decisions in natural and synthetic populations.

### Model-based inference predicts DNA damage response from division-rate selection

**Fig. 4.**
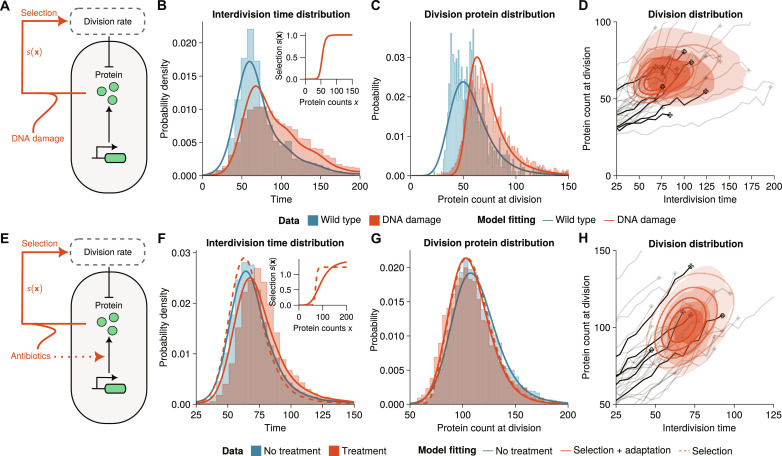
Inference of selection effects in *E. coli* cells. (**A**) Agent-based model of the SOS response involves a bursty gene expression model with an age-dependent division rate and binomial partitioning at cell division. DNA damage is induced through a gene expression–dependent modulation of the division rate. (**B** and **C**) Interdivision time and protein distributions from the mother machine lineages [fluorescent reporter, medium growth conditions; (*47*)] in unstressed (blue area) and damage-induced mother machine lineages (red area) are well fit by the agent-based model (*m* = 1; see section S4B for fit parameters). The inset shows the selection function *s*(**x**) obtained in damage-induced conditions. (**D**) The division distribution of the fitted model shows a distinctly peaked distribution (red lines) that compares well with the experimental distribution (red-shaded area) and single-cell traces of the data (representative traces shown in black and gray). (**E**) Agent-based model of antibiotic resistance gene expression using a bursty gene expression model with age-dependent division rate. Antibiotic treatment response involves positive feedback of protein expression and division through a gene expression–dependent division rate and adaption of gene expression parameters. (**F** and **G**) Statistics of the division distribution of the lineage trees [fluorescent fusion-protein genome-integrated reporter; (*36*)] in untreated (blue area) and antibiotic-treated cells (red area). Lineage tree data is well fitted by the agent-based model combining drug-dependent selection and adaptation (red solid line), but not using selection alone (red dashed line, *m* = 2, parameters in section S4B). The inset shows the corresponding selection functions *s*(**x**) obtained in treated conditions. (**H**) The division distribution under treatment shows a peaked distribution (red lines) that compares well with the experimental distribution and single-cell traces of the data (representative traces shown in black and gray).

We now apply our modeling framework to understand how cells respond to stress. To this end, we investigate the activity of the SOS pathway quantified using the SOS promoter *PsulA* driving the expression of a fluorescent reporter (*47*). Unexpectedly, we found that the expression of the reporter is highly heterogeneous even in unstressed conditions (Fig. 4C, blue-shaded bars). We use a bursty gene expression model to quantify the expression of *PsulA* (Fig. 4A and section S4B). Although we only model a single gene, burstiness describes both expression noise and upstream variability (*48*). The simple model fits the data well (Fig. 4, B and C, blue line).

In the model, we assumed the division rate to be in the form of Eq. 3, where *s*(**x**) models a damage-induced modulation and *g*(τ) is the division rate in unstressed conditions. The latter can be inferred directly from the interdivision time data of the wild type [*s*(**x**) = 1] using a nonparametric kernel density estimator (Fig. 4B, blue-shaded bars) and fits well the interdivision time distributions. To infer the reaction kinetics in unstressed conditions, we convert fluorescence measurements to molecule numbers (fig. S8) and define a likelihood function via the division distribution ν(**x**, τ) that can be maximized using Bayesian optimization [see Materials and Methods; (*49*)] to fit the reaction parameters, i.e., burst size and burst frequency.

When DNA damage was induced, cells displayed a long tail of slowly dividing cells and increased levels of SOS pathway expression (Fig. 4, B and C, red-shaded area). Intuitively, this could suggest not only up-regulation of the SOS pathway as a stress response but also positive selection on SOS levels. We investigated this by inferring stress-induced selection function *s*(**x**) (Fig. 4B, inset; see section S4B) but kept the reaction parameters as in unstressed conditions, which provided good agreement with the data (Fig. 4, B and C, red solid line). We hence conclude that up-regulation of SOS expression is not required to fit the data. We wondered whether these effects could equally be explained through a neutral model without selection but with different interdivision time distributions. The latter gave a worse fit as it overestimated the protein-interdivision time correlation (fig. S9).

Our findings thus suggest that the DNA damage response is driven mainly by division-rate selection in this system. The observation alludes to a possible bet-hedging strategy in *E. coli* where SOS expression is heterogeneous in unstressed conditions to better deal with environmental changes in stress levels (*50*).

### Antibiotic resistance gene expression involves division-rate selection and adaptation

As a second application, we considered time-lapse data of *E. coli* expressing an antibiotic resistance gene *SmR*, an efflux pump conferring resistance to streptomycin (*36*). Again, we find that protein distributions are highly heterogeneous even in the absence of antibiotics and can also be fitted by a bursty model involving a single gene (Fig. 4, E to G, blue solid line) parameterized through burst size and burst frequency.

We wondered whether antibiotic resistance gene expression represents a possible bet-hedging strategy similar to one observed for the SOS response (Fig. 4, A to D). To this end, we again modeled the division rate via Eq. 3, where now *g*(τ) is the division rate in the absence of antibiotics [*s*(**x**) = 1] and *s*(**x**) is a drug-induced modulation of division rate. We converted fluorescence to molecule numbers (fig. S10) and used Bayesian optimization to fit the protein-dependent modulation of the division rate *s*(**x**) underlying the interdivision time and protein distributions under conditions where cells have been exposed to sublethal doses of antibiotics. While this division rate described well the reduction in protein expression (Fig. 4G, red dashed line), it failed to account for the increase in interdivision time (Fig. 4F, red dashed line). Similarly, a model with no selection but matched interdivision time distributions did not fit the protein expression data (fig. S11). Intuitively, the data suggested attenuated protein expression, while cells divided slightly slower (Fig. 4, F and G, red area), which is inconsistent with the division-rate selection model.

We thus hypothesized that cells adapt gene expression during treatment. Allowing for selection on the protein as well as adjusting the rate parameters, modeling the adaptation to the new conditions, provides good agreement with the experimental data. The model fits the interdivision time (Fig. 4F, red solid line), division protein (Fig. 4G, red solid line) as well as the joint division distribution of protein count and interdivision time (Fig. 4H, red solid line). Our results suggest that burst size roughly doubles after treatment (section S4B). This highlights that adaptation plays an important role in the drug response in *E. coli*.

## DISCUSSION

We developed an agent-based framework for quantifying the feedback between stochastic gene expression, cell division, and population dynamics. Our approach allows us to derive the division distributions of lineage trees directly accessible in live-cell imaging of growing cell populations. These distributions are generally distinct from the first passage distributions of the conventional chemical master equation due to the effects of division rate, natural selection, and cell history. Our findings thus provide a quantitative understanding of how natural selection and division rates shape phenotypes and allow decision-making in response to gene expression.

Our method enables accurate solutions of agent-based models and efficient parameter inference. Previous approaches typically relied on costly agent-based simulations (*51*). To this end, we proposed a FSP solution. We identified the exit probability of crossing the state space, which serves as an error gauge and quality assurance of the numerical scheme resulting from truncating the state space and setting a finite maximum age for cells. However, the FSP method (like the master equation) presents a large system of coupled differential equations. Tensor-train methods and clever state space truncations have alleviated some of these challenges (*52*).

Multi-stable gene regulatory networks are fundamental in development, triggering cellular responses, and cell fate decisions. Naturally, these networks require environmental signals for robust decision-making, while, otherwise, they are subject to continuing fluctuations that can lead to reversible phenotypic switching. Our methodology allowed us to understand several models implementing positive feedback between protein expression and division rate. We showed that positive feedback coupled with population dynamics leads to phenotypic switching of mother machine lineages but to phenotypic selection in the histories of lineage trees, demonstrating that mother machine lineages generally do not represent typical cell lineages. Intuitively, the mother machine samples lineages with a probability that decreases exponentially with the number of divisions, thus oversampling slowly dividing cells compared to lineage trees (*36*). Division-rate selection presents a robust mechanism of irreversible lineage choice in a population. This mechanism requires slow-cycling progenitor cells as observed in stem cell lineages (*7*, *53*, *54*). Mother machine lineages and lineage tree histories, which originate from the same ancestral cell, both display phenotypic switching initially but then gradually diverge over time due to natural selection (figs. S6 and S7). This trade-off could present a strategy for adaptation to changing environments.

The present framework provides multiple advantages over previously proposed stochastic modeling approaches for single-cell data. Our framework revealed irreversible cell fate decisions that cannot be captured using traditional chemical master equation models. The agent-based approach goes beyond the snapshot statistics because it explicitly accounts for age structure. This allows us to assess lineage statistics and dynamics, such as cell birth, division events, or certain cell cycle stages. As we have demonstrated, this information is readily available from time-lapse data and allows for parameter fitting and model selection. Although we focused on division rate, growth rate is coupled to gene expression and, hence, is an important factor for selection (*9*, *25*, *55*). Our framework extends to cell size control through an effective division rate (see Materials and Methods) where selection strength is proportional to the cell growth rate. Analyzing growth and cell size dynamics could disentangle the effects of division and growth rates and provide further data insights (*56*).

Our agent-based theory provides crucial insights into how cells respond to stress and drugs based on gene expression and division feedback. To this end, we used Bayesian optimization for parameter inference on two single-cell datasets in *E. coli* (*36*, *47*). The framework allowed us to efficiently integrate data from multiple conditions to identify selection effects and evaluate competing model hypotheses. Our stochastic model suggested that the increase in SOS pathway expression in response to induced DNA damage (*47*) does not require up-regulation of SOS expression but is achieved effectively through division-rate selection. This suggests that heterogeneous SOS expression in unstressed conditions could provide a bet-hedging strategy. A similar model applied to the response of an antibiotic resistance gene revealed that division-rate selection alone was not sufficient to explain the data but that cells adapt their gene expression during treatment.

Crucially, our analysis quantified changes in gene expression and interdivision times between different experimental conditions. Yet, we cannot exclude that selection effects are present even in supposedly neutral conditions. It remains to be seen whether differences between division-rate and natural selection can be quantified from time-lapse observations of individual lineage trees. For simplicity, we assumed a multiplicative selection model, Eq. 3, where gene expression–dependent effects act independently from other sources effectively modeled through cell age. Studying the effects of cell cycle–dependent feedback on selection would be an interesting avenue for future research.

Our theory enables us to understand how variability in gene expression shapes phenotypes and propagates to population dynamics. We used the method to uncover mechanisms of cell fate decision-making, study how cells respond to stress, and adapt their gene expression programs in response to drugs. Our findings thus substantially advance the understanding of how cells make decisions through coupling gene networks with growth and division. Looking beyond gene expression, our framework could more widely be used to study phenotypic selection on other processes that affect growth, such as mitochondrial turnover (*57*). We expect our methods to provide insights into the role of heterogeneity in growth-associated diseases and drug resistance, such as in cancer (*58*).

## MATERIALS AND METHODS

### First division algorithm for exact agent-based simulation

We present an exact simulation algorithm to the agent-based model of cells in a growing population from time *t*_0_ to *T*. This is an extended version of the first division algorithm (*22*) and the Extrande thinning method for sampling division times (*59*). The algorithm uses a lookup horizon Δ*t* over which the division rate can be bounded.

1. Initialization: Initialize the cell population at time *t* = *t*_0_ by assigning each cell *i* an age τ*_i_*(*t*) at time *t* and molecule count vector **x***_i_*(τ*_i_*(*t*)).

2. Intracellular reaction dynamics: For each cell *i* simulate the trajectory of molecule counts in the age interval [τ*_i_*(*t*), τ*_i_*(*t* + Δ*t*)] using the Gillespie algorithm.

3. Cell division times: For each cell *i* sample the division time *t*_*d*,*i*_ via the thinning method.

a) Compute an upper bound of the division rate by γ_max_ ≥ γ(**x**(τ(*t*)), τ(*t*)) for all *t* in [*t*, *t* + Δ*t*].

b) Sample an event time *t*^∗^ from exponential distribution with rate γ_max_. The proposed division time is then *t*_p_ = *t* + *t*^∗^.

i. If *t*_p_ > *t* + Δ*t*, then reject the proposed division time *t*_p_ and conclude that the cell does not divide in this time interval.

ii. If *t*_p_ ≤ *t* + Δ*t*, sample a *u* from uniform distribution on the interval [0,1] and consider two options. Accept the proposed time *t*_p_ as the next division time for the cell if   $\frac{\gamma \left(x\left(\tau \left(t_{p}\right),\tau \left(t_{p}\right)\right)\right)}{\gamma_{\text{max}}}\leq u$ . Otherwise, set *t* = *t*_p_ and repeat from step 3b.

4. Next division time: Determine the next cell to divide by *j* = argmin*_i_t*_*d*,*i*_.

5. Cell division: Replace the dividing cell *j* by two daughter cells at age 0. Set the molecule counts of one by sampling from the partitioning distribution *K*_1_(**x**∣**x***_j_*(τ(*t*_*d*,*j*_))) and give the remaining molecules to the other daughter. Simulate the intracellular reaction dynamics of the two daughter cells for the time interval [*t*_*d*,*j*_, *t* + Δ*t*] and sample their division times as in step 3.

6. Repeat steps 4 and 5 until no more divisions occur in the time interval [*t*, *t* + Δ*t*]. Set *t* = *t* + Δ*t* and **x***_i_* to the molecule counts of cell *i* at time *t*. If *t* ≥ *T*, then stop; else, go to step 2.

### FSP-based solution to the agent-based model

We developed the FSP scheme for growing cell populations. FSP relies on considering the dynamics over a finite truncated state space X ⊂ *S* for **x**. The scheme effectively computes $\Pi_{n}^{\prime }\left(x|\tau \right)=\Pi_{n}\left(x|\tau \right)e^{-\int_{0}^{\tau }\mathrm{ds}\gamma \left(s\right)}$ , the product of the probability that cells have **x** molecules when they reach age τ and the probability that cells have not yet divided at that age. The cells that leave the truncated state space or do not divide in the age interval [0, τ_max_] are reinitialized at age 0 according to the division kernel *K*(**x**∣**x**′). The corresponding exit probability to be reinitialized is given by

$$\begin{array}{c} \varepsilon_{n}\left(x,\lambda_{n}\right)=\int_{0}^{\tau_{\text{max}}}d\tau \bar{q}\left(x,\tau \right)\Pi_{n}^{\prime }\left(x|\tau \right)e^{-\lambda_{n}\tau }\\ +e^{-\lambda_{n}\tau_{\text{max}}}\Pi_{n}^{\prime }\left(x|\tau_{\text{max}}\right) \end{array}$$ (4)

which can be self-consistently computed along with the FSP approximation. $\bar{q}\left(x,\tau \right)$ is the rate with which the cells with intracellular state **x** leave the truncated state space. That is, we define $\bar{q}\left(x,\tau \right)=\sum_{\bar{x}\in \bar{X}}Q_{\bar{x},x}\left(\tau \right)$ , and the complement $\bar{X}=S\setminus X$ are the intracellular states outside the truncation boundary. The first term of Eq. 4 corresponds to the probability mass leaving the population though the FSP boundary states into the complement $\bar{X}$ while second to the probability mass of cells that do not divide in the interval [0, τ_max_]. The algorithm is as follows:

1. Set *n* = 0 and provide an initial guess for the birth distribution $\Pi_{0}^{\prime }$(**x**∣0) and growth rate λ_0_.

2. Solve

$$\frac{\partial }{\partial \tau }\Pi_{n}^{\prime }\left(x|\tau \right)=\left[{\mathbb{Q}}_{X}\left(\tau \right)-\gamma (x,\tau )\right]\Pi_{n}^{\prime }\left(x|\tau \right)$$ (5)

where ${\mathbb{Q}}_{X}$ is defined as

$${\mathbb{Q}}_{X}\left(\tau \right)\Pi_{n}^{\prime }\left(x|\tau \right)=\sum_{x^{\prime }\in X}{\mathbb{Q}}_{x,x^{\prime }}\left(\tau \right)\Pi_{n}^{\prime }\left(x^{\prime }|\tau \right)$$ (6)

for all **x** in X.

3. Solve

$$\nu_{n}\left(x,\tau \right)={\mathrm{me}}^{-\lambda_{n}\tau }\gamma \left(x,\tau \right)\Pi_{n}^{\prime }\left(x|\tau \right)$$ (7)

$$1-\sum_{x\in X}\varepsilon_{n}\left(x,\lambda_{n}\right)=\int_{0}^{\tau_{\text{max}}}d\tau \sum_{x\in X}\nu_{n}\left(x,\tau \right)$$ (8)

to obtain the growth rate λ*_n_* and the division distribution ν*_n_*(**x**, τ).

4. Update the boundary condition $\Pi_{n+1}^{\prime }$(**x**∣0) using

$$\Pi_{n+1}^{\prime }\left(x|0\right)=\sum_{x^{\prime }\in X}K\left(x|x^{\prime }\right)\left[\int_{0}^{\infty }d{\tau \nu }_{n}\left(x^{\prime },\tau \right)\;+\varepsilon \left(x^{\prime },\lambda_{n}\right)\right]$$ (9)

5. Repeat from step 2 until λ*_n_* and $\Pi_{n}^{\prime }$(**x**∣0) converge.

For mother machine lineages, we have *m* = 1 and the population stays constant and, hence, λ*_n_* = 0 for all *n*. In this case, only the initial condition $\Pi_{n}^{\prime }$(**x**∣0) is iterated. The details of the derivation are given in section S1A.

### Modeling growth rate–dependent selection via effective selection strength

We consider an extended model where selection is modeled by gene expression–dependent growth and division rates. Cells grow exponentially where the cell growth rate α(**x**) is a continuous function of the gene expression state **x**. The division rate γ(**x**, ς, τ) here also depends on cell size ς, which implements common models of cell size control such as adders and sizers. The stable snapshot distribution satisfies [section S1A; see also (*60*)]

$$\left[\lambda +\frac{\partial }{\partial \tau }+\frac{\partial }{\partial \varsigma }\alpha \left(x\right)\varsigma +\gamma (x,\varsigma ,\tau )-\mathbb{Q}(\varsigma ,\tau )\right]\Pi \left(x,\varsigma ,\tau \right)=0$$ (10)

with boundary condition

$$\begin{array}{c} \Pi \left(x,\varsigma ,0\right)=m\int_{0}^{\infty }d\varsigma^{\prime }\int_{0}^{\infty }d\tau \sum_{x^{\prime }\in X}\times \\ K\left(x|x^{\prime },\frac{\varsigma }{\varsigma^{\prime }}\right)G\left(\varsigma |\varsigma^{\prime }\right)\gamma \left(x^{\prime },\varsigma^{\prime },\tau \right)\Pi \left(x^{\prime },\varsigma^{\prime },\tau \right) \end{array}$$ (11)

Here, the inherited size fraction θ has distribution ρ(θ), which influences the molecule-partitioning kernel *K*(**x**∣**x**′, θ) and also determines size partitioning via $G\left(\varsigma |\varsigma \prime \right)=\int_{0}^{1}d\theta \rho \left(\theta \right)\delta \left(\varsigma -\varsigma \prime \theta \right)$ , where δ is the Dirac delta function. This model reduces to Eqs. 1A to 1C by defining

$$\begin{array}{c} \gamma \left(x,\tau \right)=E_{\Pi }\left[\gamma \left(x,\varsigma ,\tau \right)|x,\tau \right]\\ \mathbb{Q}\left(\tau \right)=E_{\Pi }\left[\mathbb{Q}\left(\varsigma ,\tau \right)|x,\tau \right]\\ K\left(x|x^{\prime }\right)=E_{\rho }\left[K\left(x|x^{\prime },\theta \right)\right] \end{array}$$ (12)

as the effective division rate, the effective transition matrix, and the effective partitioning kernel, respectively (section S1A). Note that, for cell size–dependent propensities, this amounts to using averaged propensities *w_r_*(**x**, τ) = *E*_Π_[*w_r_*(**x**, ς, τ)∣**x**, τ] in Eq. 2.

To analyze dependence the effective division rate, we state that the division rate can be written as

γ(x,ς,τ)=α(x)ςβ(x)g(ς,τ) (13)

where, now, explicitly, α(**x**) models growth rate–dependent selection and β(**x**) models division-rate selection and *g*(ς, τ) is the cell size control (*56*, *61*). This leads to

γ(x,τ)=EΠ[γ(x,ς,τ)∣x,τ]≈s(x)g(τ) (14)

when neglecting the dependence of gene expression on cell size, i.e., $E_{\Pi }\left[\bar{\alpha }\varsigma g\left(\varsigma ,\tau \right)|x,\tau \right]\approx g\left(\tau \right)$ and $\bar{\alpha }$ denotes an arbitrary reference growth rate without selection. Hence, growth rate–dependent selection can be modeled via an effective selection strength $s\left(x\right)=\frac{\alpha \left(x\right)\beta \left(x\right)}{\bar{\alpha }}$.

### Inference from single-cell data

To perform parameter fitting for the agent-based models, we defined a log-likelihood based on *N*-independent observations of the division distribution ν(**x**, τ) as

$$\text{log}ℒ\left(\Theta \right)=\sum_{i=1}^{N}\left[\nu \left(x_{i},\tau_{i};\Theta \right)+\sum_{j=1}^{m}K(x_{i,j}|x_{i};\Theta )\right]$$ (15)

where ${\left(x_{i},{\left\{x_{i,j}\right\}}_{j=1}^{m},\tau_{i}\right)}_{i=1,\ldots ,N}$ are the observations of protein counts of mother cell at division, protein counts of the daughter cells, and interdivision times, and where Θ is the vector of parameters of the model.

Inference of reaction kinetics and division rate requires absolute quantification of protein numbers. Assuming that the partitioning of molecules between the daughter cells is binomial with parameter $\frac{1}{2}$ , a linear relationship *f_i_* = *a* ⋅ *x_i_* between fluorescence *f* and absolute protein number *x_i_* can be obtained (section S4A). In practice, one fits the relation between the partitioning variance of daughter fluorescence conditional on mother fluorescence (figs. S8 and S10). The inference problem then becomes

$$\text{log}ℒ\left(\Theta \right)=\sum_{i=1}^{N}\nu \left(f_{i}/a,\tau_{i};\Theta \right)$$ (16)

${\left(f_{i},\tau_{i}\right)}_{i=1,\ldots ,N}$ are the measured fluorescence intensity at division and the corresponding interdivision times. The details on the implementation are given in section S4C.

## Supplementary Materials

### This PDF file includes:

Sections S1 to S4 Figs. S1 to S11 Tables S1 and S2 References
