# Supplementary material for: Feedback between stochastic gene networks and population dynamics enables cellular decision-making
Source: Sci Adv. 2024 May 24;10(21):eadl4895. doi: 10.1126/sciadv.adl4895 (PMC11122677; doi:10.1126/sciadv.adl4895)
Supplement: Supplementary file 1 — Sections S1 to S4 Figs. S1 to S11 Tables S1 and S2 References [file sciadv.adl4895_sm.pdf]

Science Advances
AAAS

Supplementary Materials for

Feedback between stochastic gene networks and population dynamics enables cellular decision-making

Paul Piho and Philipp Thomas

Corresponding author: Philipp Thomas, p.thomas@imperial.ac.uk

Sci. Adv. **10**, eadl4895 (2024)
DOI: 10.1126/sciadv.adl4895

This PDF file includes:

Sections S1 to S4
Figs. S1 to S11
Tables S1 and S2
References

S1. SNAPSHOT DISTRIBUTIONS IN AGENT-BASED POPULATIONS

We consider the biochemical reactions of the form

$$\sum_{i=1}^{N_s} \nu_{ir}^- X_i \to \sum_{i=1}^{N_s} \nu_{ir}^+ X_i, \qquad r = 1, \ldots, R, \tag{S1}$$

whose dynamics are encoded in the transition matrix:

$$\mathbb{Q}_{\boldsymbol{x},\boldsymbol{x}'}(\tau) = \sum_{r=1}^{R} w_r(\boldsymbol{x}', \tau)(\delta_{\boldsymbol{x},\boldsymbol{x}'+v_r} - \delta_{\boldsymbol{x},\boldsymbol{x}'}), \tag{S2}$$

where δ is the Kronecker delta, $w_r(\boldsymbol{x}', \tau)$ are the reaction propensities, and $\nu_r = (\nu_{1r}^+ - \nu_{1r}^-, \ldots, \nu_{1r}^+ - \nu_{1r}^-)^T$ is the reaction stoichiometry. In the following we omit the age-dependence of the elements of the matrix $\mathbb{Q}(\tau)$ and simply write $q(\boldsymbol{x}, \boldsymbol{x}') = \mathbb{Q}_{\boldsymbol{x},\boldsymbol{x}'}(\tau)$.

As done in [22, 23] we derive the time-evolution of snapshot density $n(\boldsymbol{x}, \tau, t)$ corresponding to the mean number of cells with molecule counts $\boldsymbol{x}$ and age τ at time t as

$$\left(\partial_t + \partial_\tau + \gamma(\boldsymbol{x}, \tau)\right) n(\boldsymbol{x}, \tau, t) = \mathbb{Q}(\tau) n(\boldsymbol{x}, \tau, t) \tag{S3}$$

with the boundary condition

$$n(\boldsymbol{x}, 0, t) = m \int_0^\infty ds \sum_{\boldsymbol{x}' \in \mathcal{S}} dx' K(\boldsymbol{x}|\boldsymbol{x}') \gamma(\boldsymbol{x}', \tau) n(\boldsymbol{x}', \tau, t). \tag{S4}$$

The sum is taken over the countable state space $\mathcal{S}$ for the trait vector $\boldsymbol{x}$. The boundary condition corresponds to the cell divisions replacing a mother cell by m newborn daughter cells with $K(\boldsymbol{x}|\boldsymbol{x}')$ denoting the probability that a daughter cell inherits $\boldsymbol{x}$ molecules from a total of $\boldsymbol{x}'$ molecules of the mother cell. The division rate $\gamma(\boldsymbol{x}, \tau)$ depends on both the cell age as well as the cell trait vector $\boldsymbol{x}$. Letting $n(\boldsymbol{x}, \tau, t) \sim e^{\lambda t} \Pi(\boldsymbol{x}|\tau) \Pi(\tau)$, we obtain Eqs. (1). Finally $m = 2$ corresponds to the population lineage trees where each division results in two offspring while $m = 1$ corresponds to tracking a single offspring in mother machine lineages.

A. Finite State Projection method derivation

In this section we detail the construction of the finite state projection-based method for computing approximate solutions to the agent-based model. The method allows for the approximation error resulting from the truncation of the state space of the intracellular dynamics as well as error resulting from the finite time-horizon to be tracked and controlled. To that end, consider a truncated state space $\mathcal{X} \subseteq \mathcal{S}$ for the trait $\boldsymbol{x}$ and let $\bar{\mathcal{X}}$ denote the complement of $\mathcal{X}$. The evolution equations that consider only the evolution of cells that remain within the truncated state space $\mathcal{X}$ is given by the following.

$$(\partial_t + \partial_\tau + \gamma(\boldsymbol{x}, \tau)) n(\boldsymbol{x}, \tau, t) = \sum_{\boldsymbol{x}' \in \mathcal{X}} \left[q(\boldsymbol{x}, \boldsymbol{x}') n(\boldsymbol{x}', \tau, t) - q(\boldsymbol{x}', \boldsymbol{x}) n(\boldsymbol{x}, \tau, t) \right] \tag{S5}$$

$$n(\boldsymbol{x}', 0, t) = m \int_0^\infty d\tau \sum_{\boldsymbol{x}' \in \mathcal{X}} K(\boldsymbol{x}'|\boldsymbol{x}) \gamma(\boldsymbol{x}, \tau) n(\boldsymbol{x}, \tau, t) \tag{S6}$$

Let us denote

$$\mathbb{Q}_{\mathcal{X}}(\tau) n(\boldsymbol{x}, \tau, t) = \sum_{\boldsymbol{x}' \in \mathcal{X}} q(\boldsymbol{x}, \boldsymbol{x}') n(\boldsymbol{x}', \tau, t), \qquad \forall \boldsymbol{x} \in \mathcal{X}. \tag{S7}$$

In addition, we keep track of the number of cells that exit the truncated state space $\bar{\mathcal{X}}$ via the following evolution equation:

$$\partial_t m(\bar{\boldsymbol{x}}, \tau, t) = \sum_{\bar{\boldsymbol{x}} \in \bar{\mathcal{X}}} q(\bar{\boldsymbol{x}}, \boldsymbol{x}) n(\boldsymbol{x}, \tau, t). \tag{S8}$$

For brevity let us denote $\sum_{\bar{\boldsymbol{x}} \in \bar{\mathcal{X}}} q(\bar{\boldsymbol{x}}, \boldsymbol{x}) = \bar{q}(\boldsymbol{x})$. Note that the sum is taken over a potentially infinite complement $\bar{\mathcal{X}}$. However, in practice when constructing the models based on a defined stochastic reaction network we can often easily find the total rate $q(\boldsymbol{x})$ corresponding to reactions leaving the state $\boldsymbol{x} \in \mathcal{X}$. Further, we can also compute the total rate $\sum_{\boldsymbol{x}' \in \mathcal{X}} q(\boldsymbol{x}', \boldsymbol{x})$ of transitioning from $\boldsymbol{x}$ to another state within the truncation $\mathcal{X}$. We then use that $q(\boldsymbol{x}) = \sum_{\boldsymbol{x}' \in \mathcal{X}} q(\boldsymbol{x}', \boldsymbol{x}) + \bar{q}(\boldsymbol{x})$ to evaluate the $\bar{q}(\boldsymbol{x})$ without having to sum over the infinite complement $\bar{\mathcal{X}}$.

To make analytical progress we consider the asymptotic behaviour of the defined system in the regime where the population grows exponentially with doubling rate λ. In particular, $n(\boldsymbol{x}, \tau, t) \sim e^{\lambda t}\Pi(\boldsymbol{x}, \tau)$ and $m(\bar{\boldsymbol{x}}, \tau, t) \sim e^{\lambda t}\pi(\boldsymbol{x}, \tau)$ where Π and π are the snapshot densities of cells within the truncated state space and outside of it respectively. In the exponential growth regime the number of cells crossing the truncation boundary can then be given by

$$\lambda\pi(\boldsymbol{x}, \tau) = \bar{q}(\boldsymbol{x})\Pi(\boldsymbol{x}, \tau). \tag{S9}$$

The additional source of error that needs to be considered is the finite time interval for integrals involving cell age. Let us fix a finite time interval $[0, \tau_{\max}]$. The proposed method reinitialises both the cells that leave the truncation $\mathcal{X}$ and cells that do not divide in the finite time interval $[0, \tau_{\max}]$ according to the division kernel as newborn cells. Thus, we get the following system

$$(\lambda + \partial_\tau + \gamma(\boldsymbol{x}, \tau))\Pi(\boldsymbol{x}, \tau) = \mathbb{Q}_{\mathcal{X}}(\tau)\Pi(\boldsymbol{x}, \tau) \tag{S10}$$

$$\Pi(\boldsymbol{x}, 0) = m\int_0^{\tau_{\max}} d\tau \sum_{\boldsymbol{x}' \in \mathcal{X}} K(\boldsymbol{x}|\boldsymbol{x}')\left[\gamma(\boldsymbol{x}', \tau) + \frac{1}{m}\bar{q}(\boldsymbol{x}')\right]\Pi(\boldsymbol{x}', \tau) + \sum_{\boldsymbol{x}' \in \mathcal{X}} K(\boldsymbol{x}|\boldsymbol{x}')\Pi(\boldsymbol{x}', \tau_{\max}). \tag{S11}$$

We then consider the conditional distribution $\Pi(\boldsymbol{x}|\tau) = \frac{\Pi(\boldsymbol{x},\tau)}{\Pi(\tau)}$. Substituting this into the joint evolution (Equation S10) and noting that the age-dependent marginal evolution is given by

$$(\partial_\tau + \lambda + \gamma(\tau))\Pi(\tau) = 0 \tag{S12}$$

gives us

$$(\partial_\tau + \gamma(\boldsymbol{x}, \tau) - \gamma(\tau))\Pi(\boldsymbol{x}|\tau) = \mathbb{Q}_{\mathcal{X}}(\tau)\Pi(\boldsymbol{x}|\tau) \tag{S13}$$

where $\gamma(\tau)$ is the marginal division rate $\mathbb{E}_\Pi\left[\gamma(\boldsymbol{x}, \tau)|\tau\right]$ denoting the expectation taken with respect to $\Pi(\boldsymbol{x}|\tau)$. We note that

$$(\partial_\tau + \gamma(\boldsymbol{x}, \tau))\Pi'(\boldsymbol{x}|\tau) = \mathbb{Q}_{\mathcal{X}}(\tau)\Pi'(\boldsymbol{x}|\tau) \tag{S14}$$

then describes the density of cells that have not divided upon reaching the age τ leading to the first passage time distribution for a cell to divide from trait $\boldsymbol{x}$ at time τ, division distribution for brevity, given by

$$\nu(\boldsymbol{x}, \tau) = me^{-\lambda\tau}\gamma(\boldsymbol{x}, \tau)\Pi'(\boldsymbol{x}|\tau). \tag{S15}$$

Again by the definition of conditional distribution we can then derive the boundary condition for the conditional evolution as

$$\Pi(\boldsymbol{x}|0) = \sum_{\boldsymbol{x}' \in \mathcal{X}} K(\boldsymbol{x}|\boldsymbol{x}')\left[\int_0^{\tau_{\max}} d\tau\left[\nu(\boldsymbol{x}', \tau) + e^{-\lambda\tau}\bar{q}(\boldsymbol{x}')\Pi'(\boldsymbol{x}'|\tau)\right] + e^{-\lambda\tau_{\max}}\Pi'(\boldsymbol{x}'|\tau_{\max})\right]. \tag{S16}$$

The exit probability

$$\varepsilon(\boldsymbol{x}', \lambda) = \int_0^{\tau_{\max}} d\tau e^{-\lambda\tau}\bar{q}(\boldsymbol{x}')\Pi'(\boldsymbol{x}'|\tau) + e^{-\lambda\tau_{\max}}\Pi'(\boldsymbol{x}'|\tau_{\max}) \tag{S17}$$

corresponds to the cells leaving the truncation from state $\boldsymbol{x}'$ and not dividing within the finite time horizon $[0, \tau_{\max}]$. Note that summing over $\boldsymbol{x}' \in \mathcal{X}$ corresponds to the total exit probability ε for a cell to leave the state space

$$\varepsilon(\lambda) = \int_0^{\tau_{\max}} d\tau e^{-\lambda\tau}\sum_{\boldsymbol{x} \in \mathcal{X}} \bar{q}(\boldsymbol{x})\Pi'(\boldsymbol{x}|\tau) + e^{-\lambda\tau_{\max}}\sum_{\boldsymbol{x} \in \mathcal{X}} \Pi'(\boldsymbol{x}|\tau_{\max}). \tag{S18}$$

This takes into account truncation as well as the finite time-horizon used to compute the transient evolution of $\Pi'(\boldsymbol{x}|\tau)$. Summing the boundary condition over $\boldsymbol{x}$ then gives

$$1 = m\int_0^{\tau_{\max}} d\tau e^{-\lambda\tau}\nu(\tau) + \varepsilon(\lambda) \tag{S19}$$

In the solution algorithm, presented in the Methods section of the main text, the equations (S16) and (S19) are computed at every iteration step. The implementation of the solution algorithm makes use of the Julia FiniteStateProjection package [62] for the construction of truncated $\mathbb{Q}(\tau)$.

Note that if $\varepsilon(\boldsymbol{x}, \lambda) = 0$ for all $\boldsymbol{x} \in \mathcal{X}$ then the boundary condition for the population lineage trees described in the main text by Equations (1) is recovered. Moreover

$$1 = m \int_0^\infty d\tau e^{-\lambda\tau} \nu(\tau) \tag{S20}$$

would correspond to the Euler-Lotka equation for the population lineage trees in the case of the untruncated state space and the time horizon $[0, \infty]$.

S2. RELATING GROWTH RATE SELECTION TO DIVISION RATE SELECTION

Here, we extend the snapshot density considered in Section S1 to cell size dynamics with growth-rate mediated selection. In particular, we assume that exponential growth of cell size ς with growth rate $\alpha(\boldsymbol{x})$, which continuously depends on the molecule numbers $\boldsymbol{x}$. We arrive at the following evolution equation for the snapshot density

$$\left(\partial_t + \partial_\tau + \partial_\varsigma \alpha(\boldsymbol{x})\varsigma + \gamma(\boldsymbol{x}, \varsigma, \tau)\right) n(\boldsymbol{x}, \varsigma, \tau) = \mathbb{Q}(\tau, \varsigma) n(\boldsymbol{x}, \varsigma, \tau) \tag{S21}$$

with boundary condition

$$n(\boldsymbol{x}, \varsigma, 0) = m \int_0^\infty d\varsigma' \int_0^\infty d\tau \sum_{\boldsymbol{x}' \in \mathcal{X}} K(\boldsymbol{x}|\boldsymbol{x}', \frac{\varsigma}{\varsigma'}) G(\varsigma|\varsigma') \gamma(\boldsymbol{x}', \varsigma', \tau) n(\boldsymbol{x}', \varsigma', \tau). \tag{S22}$$

The partitioning of the molecule numbers is now assumed to depend also on the proportion of the size $\frac{\varsigma}{\varsigma'}$ a daughter cell inherits from the mother cell of size ς'. The $G(\varsigma|\varsigma')$ defines the probability of a cell with size ς' at division ending up with size ς after division. For example, for independent binomial partitioning, each molecule is partitioned into a daughter with a probability θ equal to the inherited size fraction, i.e.,

$$K(\boldsymbol{x}|\boldsymbol{x}', \theta) = \prod_{i=1}^{N_s} \binom{x_i'}{x_i} \theta^{x_i} (1-\theta)^{x_i' - x_i}. \tag{S23}$$

The inherited size fraction also defines the size division kernel:

$$G(\varsigma|\varsigma') = \int_0^1 d\theta \rho(\theta) \delta(\varsigma - \varsigma'\theta), \tag{S24}$$

where δ is the Dirac delta function and ρ satisfies $\rho(\theta) = \frac{1}{2}\bar{\rho}(\theta) + \frac{1}{2}\bar{\rho}(1-\theta)$ with $\bar{\rho}$ modelling the inherited size fraction accounting asymmetric cell division. The snapshot distribution in the asymptotic limit $t \to \infty$ where the population grows exponentially with growth rate λ is then given by

$$\left(\lambda + \partial_\tau + \partial_\varsigma \alpha(\boldsymbol{x})\varsigma + \gamma(\boldsymbol{x}, \varsigma, \tau)\right) \Pi(\boldsymbol{x}, \varsigma, \tau) = \mathbb{Q}(\tau, \varsigma) \Pi(\boldsymbol{x}, \varsigma, \tau) \tag{S25}$$

with boundary condition

$$\Pi(\boldsymbol{x}, \varsigma, 0) = m \int_0^\infty d\varsigma' \int_0^\infty d\tau \sum_{\boldsymbol{x}' \in \mathcal{X}} K(\boldsymbol{x}|\boldsymbol{x}', \frac{\varsigma}{\varsigma'}) G(\varsigma|\varsigma') \gamma(\boldsymbol{x}', \varsigma', \tau) \Pi(\boldsymbol{x}', \varsigma', \tau). \tag{S26}$$

Integrating the division kernel over the birth sizes ς

$$\int_0^\infty d\varsigma K(\boldsymbol{x}|\boldsymbol{x}', \frac{\varsigma}{\varsigma'}) G(\varsigma|\varsigma') = \int_0^\infty d\varsigma K(\boldsymbol{x}|\boldsymbol{x}', \frac{\varsigma}{\varsigma'}) \int_0^1 d\theta \rho(\theta) \delta(\varsigma - \varsigma'\theta) = \mathbb{E}_\rho \left[K(\boldsymbol{x}|\boldsymbol{x}', \theta) \right] \tag{S27}$$

Using the law of conditional probability $\Pi(\boldsymbol{x}, \varsigma, \tau) = \Pi(\varsigma|\boldsymbol{x}, \tau)\Pi(\boldsymbol{x}, \tau)$ and marginalising out ς, the evolution equation for the marginal snapshot distribution with boundary condition becomes

$$\left(\partial_\tau + \lambda + \mathbb{E}_\Pi \left[\gamma(\boldsymbol{x}, \varsigma, \tau)|\boldsymbol{x}, \tau\right]\right) \Pi(\boldsymbol{x}, \tau) = \mathbb{E}_\Pi \left[\mathbb{Q}(\tau, \varsigma)|\boldsymbol{x}, \tau\right] \Pi(\boldsymbol{x}, \tau) \tag{S28}$$

$$\Pi(\boldsymbol{x}, 0) = m \int_0^\infty d\tau \sum_{\boldsymbol{x}' \in \mathcal{X}} \mathbb{E}_\rho \left[K(\boldsymbol{x}|\boldsymbol{x}', \theta)\right] \mathbb{E}_\Pi \left[\gamma(\boldsymbol{x}', \varsigma', \tau)|\boldsymbol{x}', \tau\right] \Pi(\boldsymbol{x}', \tau) \tag{S29}$$

We define

$$\mathbb{Q}(\tau) = \mathbb{E}_\Pi[\mathbb{Q}(\tau,\varsigma)|\boldsymbol{x},\tau], \tag{S30}$$
$$\gamma(\boldsymbol{x},\tau) = \mathbb{E}_\Pi\left[\gamma(\boldsymbol{x},\varsigma,\tau)|\boldsymbol{x},\tau\right], \tag{S31}$$
$$K(\boldsymbol{x}|\boldsymbol{x}) = \mathbb{E}_\rho\left[K(\boldsymbol{x}|\boldsymbol{x},\theta)\right] \tag{S32}$$

as the marginal division rate and the effective division kernel, respectively. We then recover the model that depends only on the gene expression state $\boldsymbol{x}$ and the cell age τ:

$$\left(\partial_\tau + \lambda + \gamma(\boldsymbol{x},\tau)\right)\Pi(\boldsymbol{x},\tau) = \mathbb{Q}(\tau)\Pi(\boldsymbol{x},\tau) \tag{S33}$$
$$\Pi(\boldsymbol{x},0) = m\int_0^\infty d\tau \sum_{\boldsymbol{x}'\in\mathcal{X}} K(\boldsymbol{x}|\boldsymbol{x}')\gamma(\boldsymbol{x}',\tau)\Pi(\boldsymbol{x}',\tau). \tag{S34}$$

The model without cell size control is thus a reduced model of the developed cell size control model.

S3. MODEL DESCRIPTIONS

A. Telegraph model

In this section we provide the complete specification of the agent-based telegraph model used in the main manuscript. The intracellular dynamics are given by the following stochastic reaction network

$$G_{off} \xrightarrow{k_{on}} G_{on}$$
$$G_{on} \xrightarrow{k_{off}} G_{off}$$
$$G_{on} \xrightarrow{k_{prod}} G_{on} + b \times x$$
$$x \xrightarrow{k_{deg}} \emptyset$$

where $b \sim Geometric(\bar{b})$ is a geometrically distributed random variable parametrised by the mean $\bar{b}$. In practice, to simplify the implementation with Julia Catalyst package [63] the bursty reaction was truncated and split into multiple reactions

$$G_{on} \xrightarrow{p(1)k_{prod}} G_{on} + 1 \times x$$
$$\dots$$
$$G_{on} \xrightarrow{p(N)k_{prod}} G_{on} + N \times x$$

where the rate of each reaction is weighed by the probability density p of the burst size. Note that the truncation of the bursty reaction is not necessary for the computations of $\bar{q}$ in Section S1 A. The total rate out of the each state with gene on and protein count x is the sum of k_{off}, k_{deg} and k_{prod}. Thus all we need to find the rate corresponding to cells leaving the truncation is to consider the cumulative probability of bursts that do not leave the truncation.

In the case of neutral condition with no selection the division rate is given by $\gamma(\boldsymbol{x},\tau) = g(\tau)$ where $g(\tau)$ is the hazard of the Gamma distribution parametrised by mean its μ and squared coefficient of variation cv^2. In general, if f and F are the probability density and cumulative distribution functions of a distribution respectively, then the hazard of the distribution is defined by

$$g(\tau) = \frac{f(\tau)}{1 - F(\tau)}. \tag{S35}$$

In the selection case the division rate is given by $\gamma(\boldsymbol{x},\tau) = s(\boldsymbol{x})g(\tau)$ where $s(x)$ is a Hill function

$$s(\boldsymbol{x}) = \frac{1}{\left(\frac{K}{x}\right)^n + 1}. \tag{S36}$$

The parameters are chosen to describe the slow-switching dynamics of the promoter state to give rise to bimodal expression levels and are given in the table below.

k_{on}	k_{off}	k_{prod}	mean burst size $\bar{b}$	k_{deg}	$Gamma(\mu, cv^2)$	Hill exponent n	Hill coefficient K
0.7	0.2	20.0	1.2	0.1	$(0.8595, 0.6)$	2	4.472

B. Single gene feedback models

There are several models that follow the same general structure presented in the main text. We start by presenting the general structure of the agent-based and effective dilution formulations of the single gene feedback models. The specific instances of these models are then arrived to by choosing different ways to define the rate functions within the models.

1. Agent-based model

The intracellular dynamics of the agent-based model are given by the following general stochastic reaction network

$$\emptyset \xrightarrow{r_{prod}(\vec{x})} x \qquad\qquad x \xrightarrow{r_{deg}(\vec{x})} \emptyset$$

modelling production and degradation of a single protein x with rates $r_{prod}(\vec{x})$ and $r_{deg}(\vec{x})$ respectively. The general model assumes the division rate $\gamma(\boldsymbol{x}, \tau)$ is in the form

$$\gamma(\boldsymbol{x}, \tau) = s(\boldsymbol{x})g(\tau).$$

The partitioning of mother cell protein numbers at division follows symmetric binomial partitioning.

2. Effective dilution model

In the effective dilution model we add a linear dilution term to the stochastic reaction network

$$\emptyset \xrightarrow{r_{prod}(\vec{x})} x \qquad\qquad x \xrightarrow{r_{dil}(\vec{x})+r_{deg}(\vec{x})} \emptyset.$$

where $r_{dil}(\vec{x})$ is the rate of dilution modelling the loss of protein due to cell divisions in an exponentially growing population. A common approach when no division-rate selection on x is considered is to take $r_{dil}(\vec{x}) = \lambda$ where λ is the doubling time of the cell population. The stationary solutions for the birth-death processes arising from the effective dilution models were computed using standard steady state methods [64].

3. Model instances

There are three different variants of the single protein feedback model that are considered in this paper. Each of them use the same general outline defined in Sections S3 B 1 and S3 B 2.

a. Transcriptional feedback model The transcriptional feedback model considers the protein x promoting its own production without division-rate selection on x by defining the rate of production and degradation as

$$r_{prod}(\boldsymbol{x}) = \alpha + k/((K/x)^2 + 1)) \qquad\qquad r_{deg}(\boldsymbol{x}) = 0. \tag{S37}$$

The degradation rate of the protein is taken to be 0 for simplicity. This model considers no division-rate selection on x and thus we take $s(\boldsymbol{x}) = 1$. The age-dependent division-rate component $g(\tau)$ is given as the hazard of the Gamma distribution parametrised by its mean μ and the squared coefficient of variation cv^2. Let f and F denote the probability density and cumulative distribution functions of the Gamma distribution respectively. The division rate $\gamma(\boldsymbol{x}, \tau)$ is then given by

$$\gamma(\boldsymbol{x}, \tau) = \frac{f(\tau)}{1 - F(\tau)}. \tag{S38}$$

The dilution rate in the effective dilution model is defined as

$$r_{dil}(\boldsymbol{x}) = \lambda \tag{S39}$$

where λ is the doubling time of an exponentially growing cell population resulting from the Gamma distributed inter-division time distribution above. The parametrisations chosen to give rise to bimodal behaviour are given in the table below.

α	k	Hill coefficient	K	λ	$Gamma(\mu, cv^2)$
$\{0.0, 0.5, \cdots, 7\}$	60.0		31.62	1.0	$(0.7177, 0.1)$

b. Positive growth feedback model The positive growth feedback model uses the general structure outline in Sections S3 B 1 and S3 B 2 and features a constant production and degradation rate

$$r_{prod}(\boldsymbol{x}) = \alpha \qquad r_{deg}(\boldsymbol{x}) = \delta.$$

The x-dependent division rate function $s(\boldsymbol{x})$ is given by a repressive Hill function

$$s(\boldsymbol{x}) = k/((x/K)^3 + 1).$$

while the age-dependent component is taken to be constant $g(\tau) = 1.0$ corresponding to exponential division time distribution when no selection on gene expression is considered. The dilution rate in the effective dilution model is defined as

$$r_{dil}(\boldsymbol{x}) = k/((x/K)^3 + 1).$$

The parametrisations of the model are given in the table below.

α	k	Hill coefficient	K	δ
$\{15, 20, \cdots, 60\}$	20.0		4.64	1.0

c. Combined feedback model The combined feedback model combines the transcriptional feedback mechanism from Section S3 B 3 a and growth feedback mechanism from Section S3 B 3 b. In particular,

$$r_{prod}(\boldsymbol{x}) = \alpha + k_1/((K_1/x)^2 + 1) \qquad r_{deg}(\boldsymbol{x}) = \delta.$$

The division rate function is similar to growth feedback model (with higher Hill exponent) and is given by a Hill function dependent on the protein counts

$$s(\boldsymbol{x}) = k_2/((x/K_2)^4 + 1) \qquad g(\tau) = 1.$$

In the effective dilution model consider the corresponding dilution term

$$r_{dil}(\boldsymbol{x}) = k_2/((x/K_2)^4 + 1)$$

The parametrisations chosen to give rise to bimodal behaviour are given in the table below.

α	k_1	Hill coefficient	K_1	k_2	Hill coefficient	K_2	δ
$\{5.88, 6.02, \cdots, 7.00\} \times 10^2$	5.6×10^3	140.0		40.0	16.46		25.0

C. Genetic toggle switch

Here we provide the complete specifications of the genetic toggle switch model used in the main manuscript.

1. *Agent-based model*

The intracellular dynamics of the agent-based model are given by the following stochastic reaction network

$$\emptyset \xrightarrow{r_{prodA}(\boldsymbol{x})} A \qquad A \xrightarrow{r_{degA}(\boldsymbol{x})} \emptyset$$

$$\emptyset \xrightarrow{r_{prodB}(\boldsymbol{x})} B \qquad B \xrightarrow{r_{degB}(\boldsymbol{x})} \emptyset$$

where the functions defining the rates of production and degradation for the two protein counts x_A and x_B are as follows:

$$r_{prodA}(\boldsymbol{x}) = k_0 + k_1/((K_1/x_B)^2 + 1) \qquad r_{degA}(\boldsymbol{x}) = \delta$$
$$r_{prodB}(\boldsymbol{x}) = \alpha/((K_2/x_A)^2 + 1) \qquad r_{degB}(\boldsymbol{x}) = \delta.$$

In particular, the proteins A and B inhibit each other's expression. We call α the induction strength. The division rate $\gamma(\boldsymbol{x}, \tau)$ for the agent-based model is then given by

$$\gamma(\boldsymbol{x}, \tau) = s(\boldsymbol{x})g(\tau).$$

where

$$s(\boldsymbol{x}) = k_3/((x_A/K_3)^2 + 1) \qquad g(\tau) = 1.0$$

In particular, we are considering positive division-rate selection on the protein A.

2. *Effective dilution model*

As previously, in the effective dilution model we add a linear dilution term to the stochastic reaction network

$$\emptyset \xrightarrow{r_{prodA}(\boldsymbol{x})} A \qquad A \xrightarrow{r_{dil}(\boldsymbol{x}) + r_{degA}(\boldsymbol{x})} \emptyset$$
$$\emptyset \xrightarrow{r_{prodB}(\boldsymbol{x})} B \qquad B \xrightarrow{r_{dil}(\boldsymbol{x}) + r_{degB}(\boldsymbol{x})} \emptyset$$

where $r_{dil}(\boldsymbol{x})$ is the rate of dilution modelling the loss of protein due to cell divisions in an exponentially growing population. The dilution rate in the effective dilution model is defined as

$$r_{dil}(\boldsymbol{x}) = k_3/((x_A/K_3)^2 + 1).$$

The parametrisations of the model are given in the table below.

α	k_0	k_1	K_1	K_2	δ	k_3	K_3
$\{16.0, 16.5, \cdots, 22.0\}$	0.4	20.0	20.0	20.0	0.2	4.0	120.0

S4. PARAMETER AND DIVISION RATE INFERENCE

A. Conversion factor

The *Escherichia coli* datasets [36, 47] analysed in the main text report fluorescence intensity of the reporter protein. In order to fit the agent-based model to data we convert the intensity to copy numbers of the protein by analysing the variability in the mother and daughter cell fluorescences at division as done, for example, in [65]. To that end we find a linear relationship $f = ax$ between the copy numbers of fluorescent molecules x and fluorescence intensity f. This conversion is done under the assumption that partitioning of the fluorescent molecules between the two daughter cells at division is binomial with probability $p = \frac{1}{2}$. Variance of the daughter cell fluorescence f_d is then given by

$$Var(f_d) = Var(ax_d) = a^2 p(1-p)x_m = \frac{a}{4} f_m$$

where x_m and f_m are the molecule count and fluorescence of the mother cell respectively. From the data we then fit the slope $\bar{a}$ of $Var(f_d) = \bar{a} f_m$ and equate it with $\frac{a}{4}$ to estimate the conversion factor a.

B. Bursty gene expression model

The agent-based model considered for both the DNA damage response and antibiotic resistance data sets is the same with different parametrisations. The intracellular dynamics of the agent-based model are given by the following stochastic reaction network

$$\emptyset \xrightarrow{k} b \times x$$

where $b \sim \mathit{Geometric}(\bar{b})$ is a geometrically distributed random variable parametrised by the mean $\bar{b}$ (see Section S3 A for implementation details). The division rate of the model is given by $\gamma(\boldsymbol{x}, \tau) = s(\boldsymbol{x})g(\tau)$ where $g(\tau)$ is the hazard of the kernel density estimate of the interdivision times in the data. If f and F denote the probability density and cumulative distribution functions of the kernel density estimate

$$g(\tau) = \frac{f(\tau)}{1 - F(\tau)}. \tag{S40}$$

The function $s(\boldsymbol{x})$ is fitted to individual conditions. In the cases of no antibiotic treatment and no induced DNA damage we assume there is no division-rate selection on the gene expression and thus the $s(\boldsymbol{x}) = 1$. In the cases of antibiotic treatment and induced DNA damage we fit the parameters of a Hill function of the form

$$s(\boldsymbol{x}) = \frac{L(1-\delta)x^m}{(K^m + x^m) + \delta}. \tag{S41}$$

The partitioning of mother cell molecule numbers at division follows symmetric binomial partitioning. The parameters fitted for the DNA damage response model is given in Table S1 and the parameters fitted for the antibiotic resistance model are given in Table S2.

	k	b	L	K	δ	m
Wild type	0.2070	1.4643	N/A	N/A	N/A	N/A
DNA damage	0.2070	1.4643	1.0469	44.68	0.0	11

TABLE S1. The parameters fitted for the DNA damage response model via Bayesian Optimisation.

	k	b	L	K	δ	m
No treatment	0.5684	1.4087	N/A	N/A	N/A	N/A
Treatment (selection)	0.5684	1.4087	1.2336	70.4	0.0	20
Treatment (selection + adaptation)	0.3378	2.0725	1.4643	92.8	0.0	4

TABLE S2. The parameters fitted for the antibiotic resistance model via Bayesian Optimisation.

C. Bayesian optimization

The Bayesian optimisation routine uses the `scikit-optimize` Python package with the default options [66]. In particular, the optimiser uses the Matérn kernel with smoothness parameter $\nu = 2.5$ corresponding to twice differentiable functions. The length scales of the kernel are tuned by the optimising routine by maximising the log marginal likelihood. Finally, the acquisition function is chosen probabilistically at every iteration between the lower confidence bound, negative expected improvement and negative probability improvement acquisition functions implemented in the package. We performed the parameter optimisation with 200 evaluations of the likelihood function and initialised with 10 random parametrisations. This was replicated 5 times with the optimal parametrisation across the replications chosen. The bounds used to constrain the search space are given in the table below.

k	b	L	K	δ	m
(0.0, 1.0)	(1.0, 10.0)	(0.0, 5.0)	(0.0, 150.0)	(0.0, 1.0)	(0, 20)

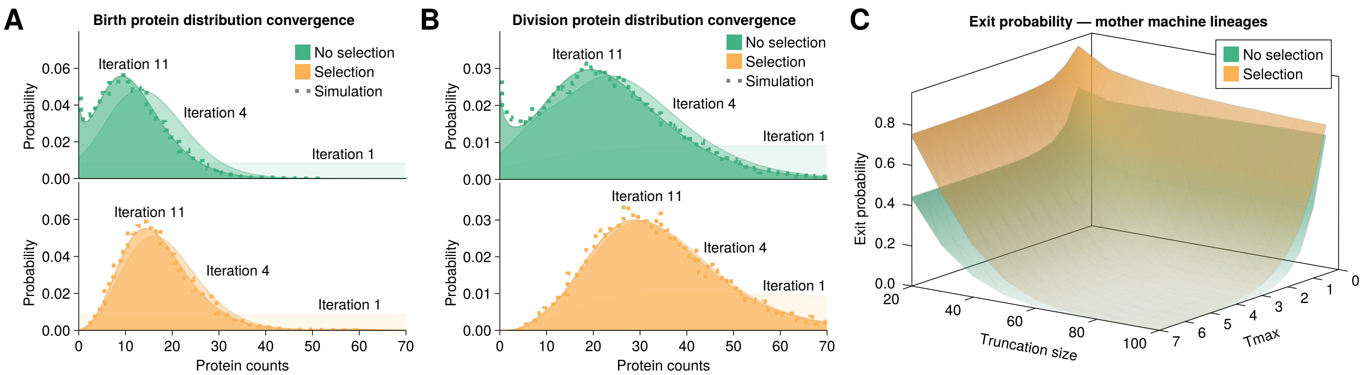

FIG. S1. Convergence of the mother machine lineage statistics of the telegraph model. **(A-B)** Birth (panel **A**) and division (panel **B**) distributions of the mother machine lineage model with fixed a truncation size and $\tau_{\max}$ converges in a couple of iterations and agrees with the agent-based simulation. **(C)** The exit probability due to the FSP truncation decreases with the truncation size and time horizon $\tau_{\max}$.

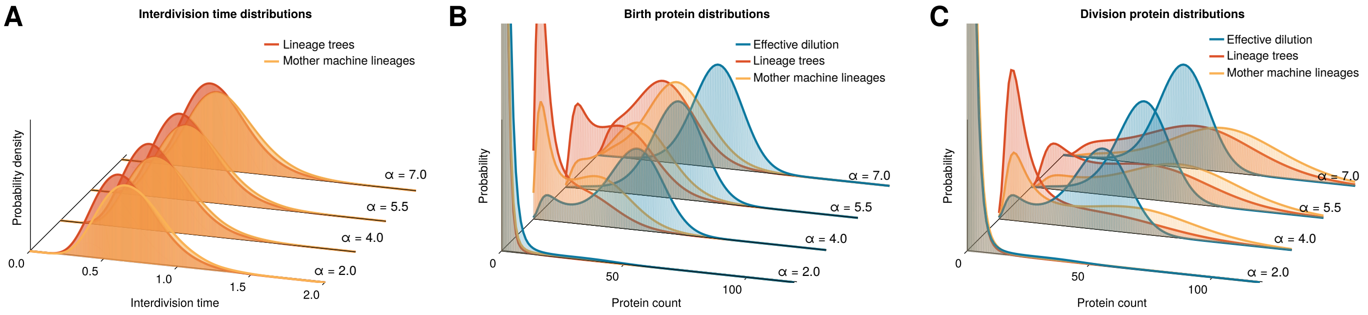

FIG. S2. Summary distributions of the transcriptional feedback model. **(A)** The interdivision time distribution shows slow dividing cells being over-represented in mother machine lineages (yellow) compared to population lineage trees (red). **(B-C)** Protein distributions predicted by the agent-based models at birth (panel **B**) and division (panel **C**) are compared with the stationary distribution of the effective dilution model.

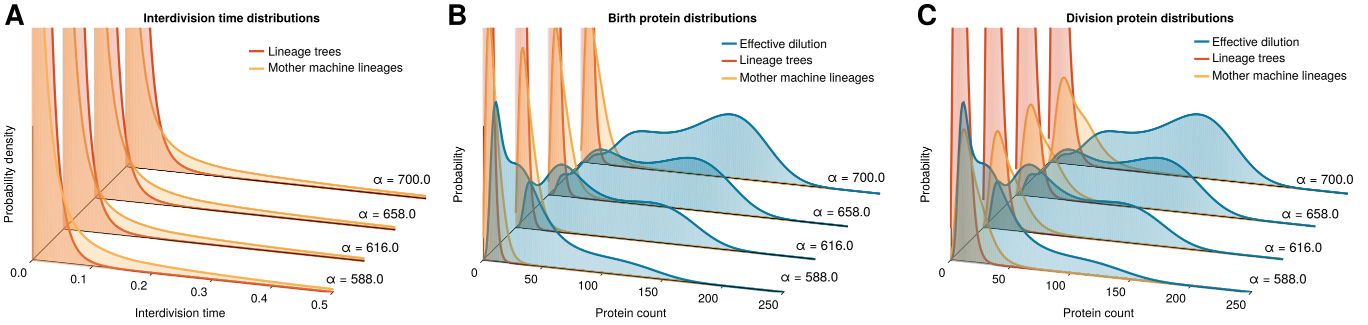

FIG. S3. Summary distributions of the model combining transcriptional and growth feedback mechanism. **(A)** The interdivision time distributions display the behaviour observed for the growth feedback model with the right tail of the unimodal interdivision time distribution becoming longer as the synthesis rate α increases. As before the corresponding slow dividing cells are repressed in the lineage tree statistics. **(B-C)** Protein distributions predicted by the agent-based models at birth (panel **B**) and division (panel **C**) are compared with the stationary distribution of the effective dilution model.

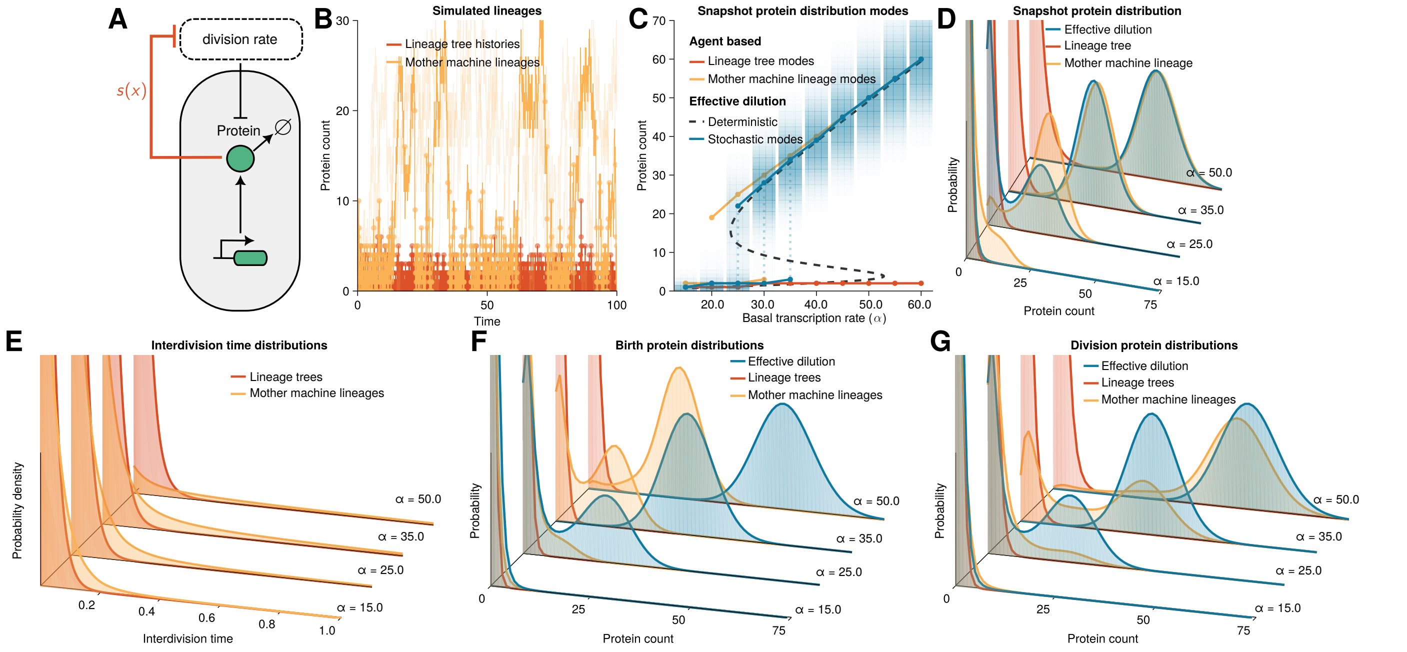

FIG. S4. **(A-D)** Agent-based model with growth feedback (SI Section S3 B 3 b). **(A)** Illustration of the growth feedback model where stable proteins are synthesized with rate α and high protein abundance inhibits cell divisions. **(B)** Agent-based simulations of mother machine lineages ($\alpha = 20.0$) show switching between low and high protein levels while, in lineage tree histories, the fast dividing lineages determine the cell fate. **(C)** Protein distribution modes display bimodality for mother machine lineages and EDM but only one mode for lineage trees. **(D)** Protein distribution of mother machine lineages display slowly diving subpopulations not seen in lineage trees. **(E)** The interdivision time distribution shows slow dividing cells are over-represented in mother machine lineages (yellow) compared to the population lineage trees (red). The right tail of the unimodal interdivision time distribution corresponding to the mother machine lineages becomes longer as the protein production rate α increases. The corresponding slow dividing cells are repressed in the lineage tree statistics. **(F-G)** Protein distributions predicted by the agent-based models at birth (panel **F**) and division (panel **G**) are compared with the stationary distribution of the effective dilution model.

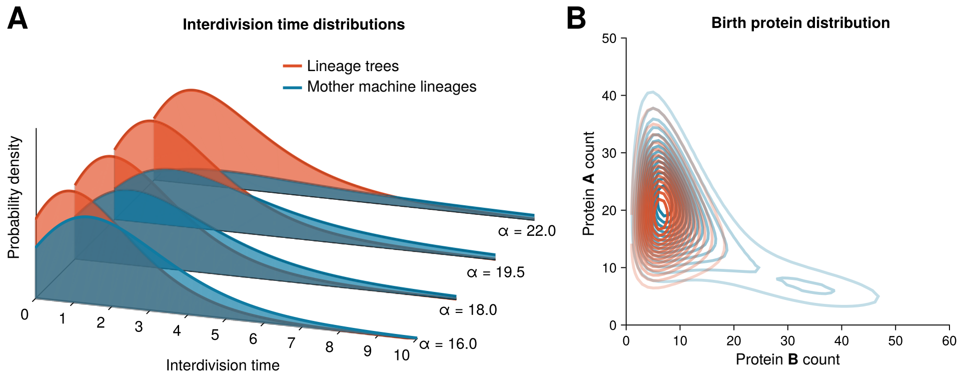

FIG. S5. Summary distributions of the genetic toggle switch (SI S3 C). **(A)** The right tail of the unimodal interdivision time distributions becomes longer as the synthesis rate α increases. The corresponding slow dividing cells are repressed in the lineage tree statistics compared to the mother machine lineages. **(B)** Birth protein distributions predicted by the agent-based models ($\alpha = 18.9$) for mother machine lineages (blue) displays bimodality with the less prominent peak corresponding to slow-dividing cells while in lineage trees (red) this peak is missing.

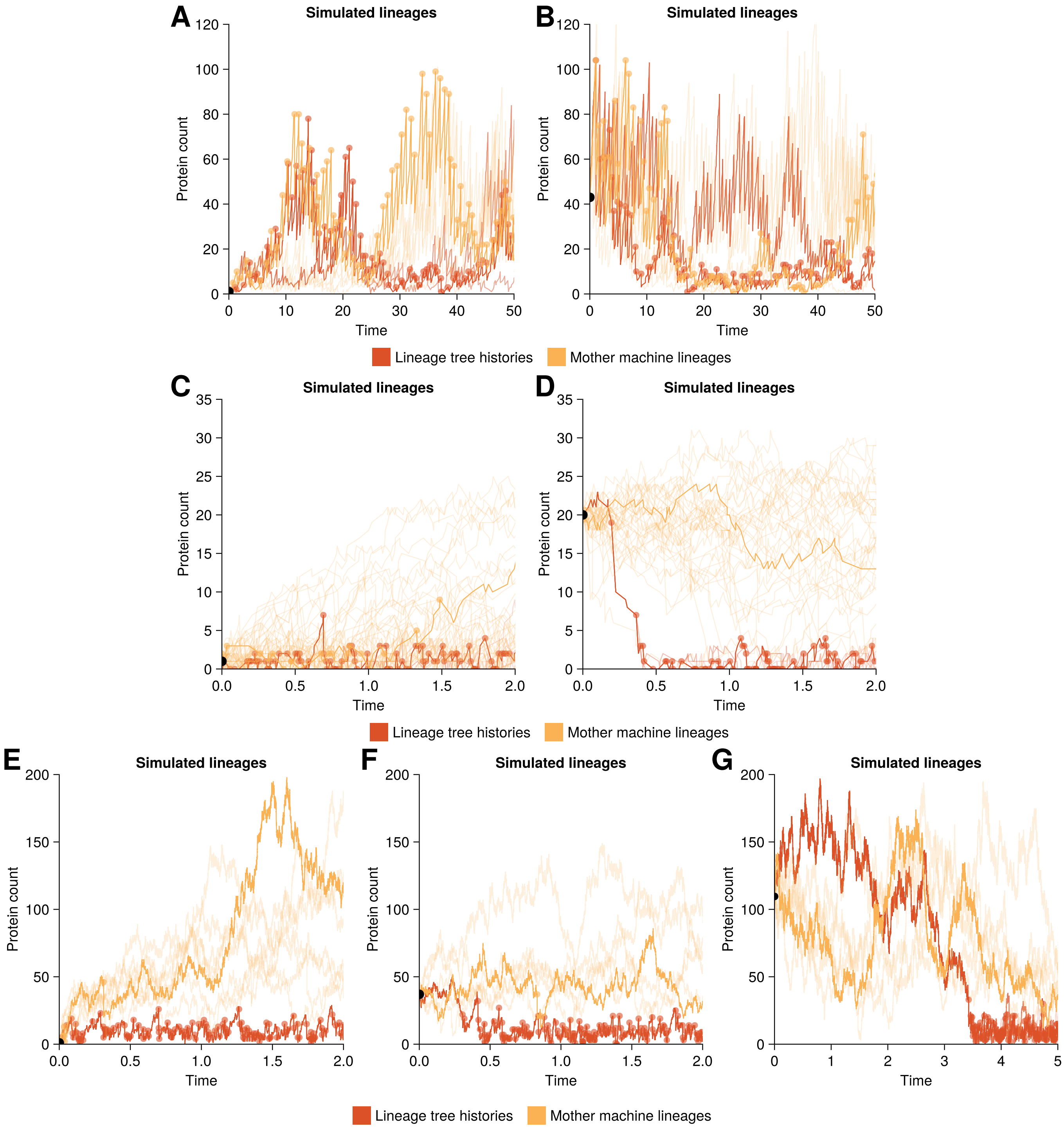

FIG. S6. Agent-based stochastic simulations of lineage tree histories (red) and mother cell lineages (yellow) of the single gene feedback models. **(A-B)** Simulations of transcriptional feedback model (model details in SI Section S3 B 3 a) for $\alpha = 4.0$. Agent-based simulations with cells initialised in a low expression level state (1 molecule, panel **A**) and a high expression level state (20 molecule, panel **B**). Lineage tree histories and mother machine lineages explore both the high expression and low expression states under both initialisations. **(C-D)** Simulations of the growth feedback model (model details in SI Section S3 B 3 b) for $\alpha = 20.0$. **(C)** Agent-based simulations initialised with cells in a low expression level fast dividing state (1 molecule). The lineage tree histories remain in their initial fast dividing state while mother machine lineages eventually visit the slow dividing state. **(D)** Agent-based simulations initialised with cells in a high expression level state (20 molecules). Lineage tree histories show strong selection for the fast dividing cell lineages in a population with histories coming down to the low expression fast dividing state in a couple of divisions. The histories resulting from cells that quickly make the transition to fast dividing regime are going to be highly represented in lineage tree histories. **(E-G)** Simulations of the combined feedback model (model details in SI Section S3 B 3 c) for $\alpha = 616.0$. Agent-based simulations with cells initialised in a low expression level state (1 molecule, panel **E**) and two high expression level states (37 molecules in panel **F**, 111 molecules in panel **G**). On short time scales the mother machine lineages and lineage tree histories agree with the qualitative dynamics of each other. However, over time the lineage tree histories settle in the fast dividing cell state.

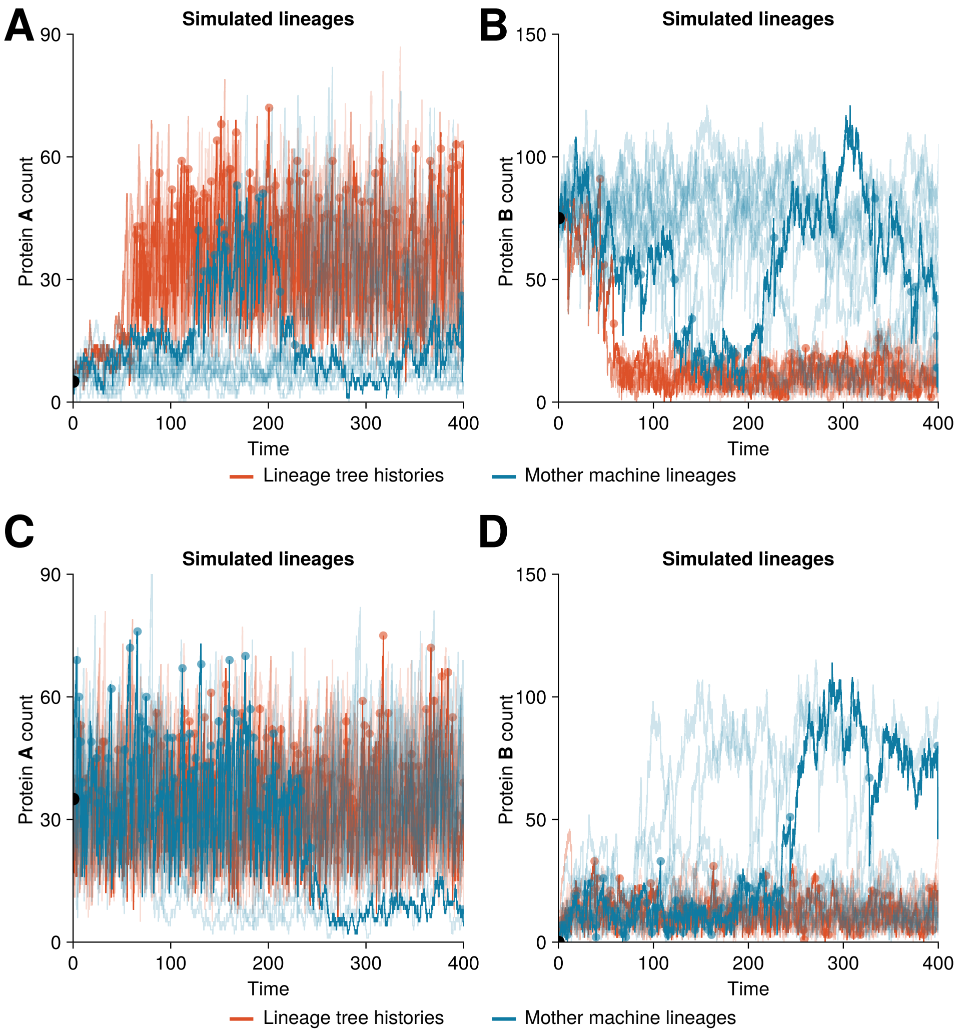

FIG. S7. Agent-based stochastic simulations of lineage tree histories (red) and mother cell lineages (blue) of the genetic toggle switch for induction strength $\alpha = 18.9$ (model details in SI S3 C). **(A-B)** Simulations trajectories starting with a cell in a slow dividing state (protein A count 5, B count 75). **(C-D)** Simulations trajectories starting with a cell in a fast dividing state (protein A count 35, B count 0).

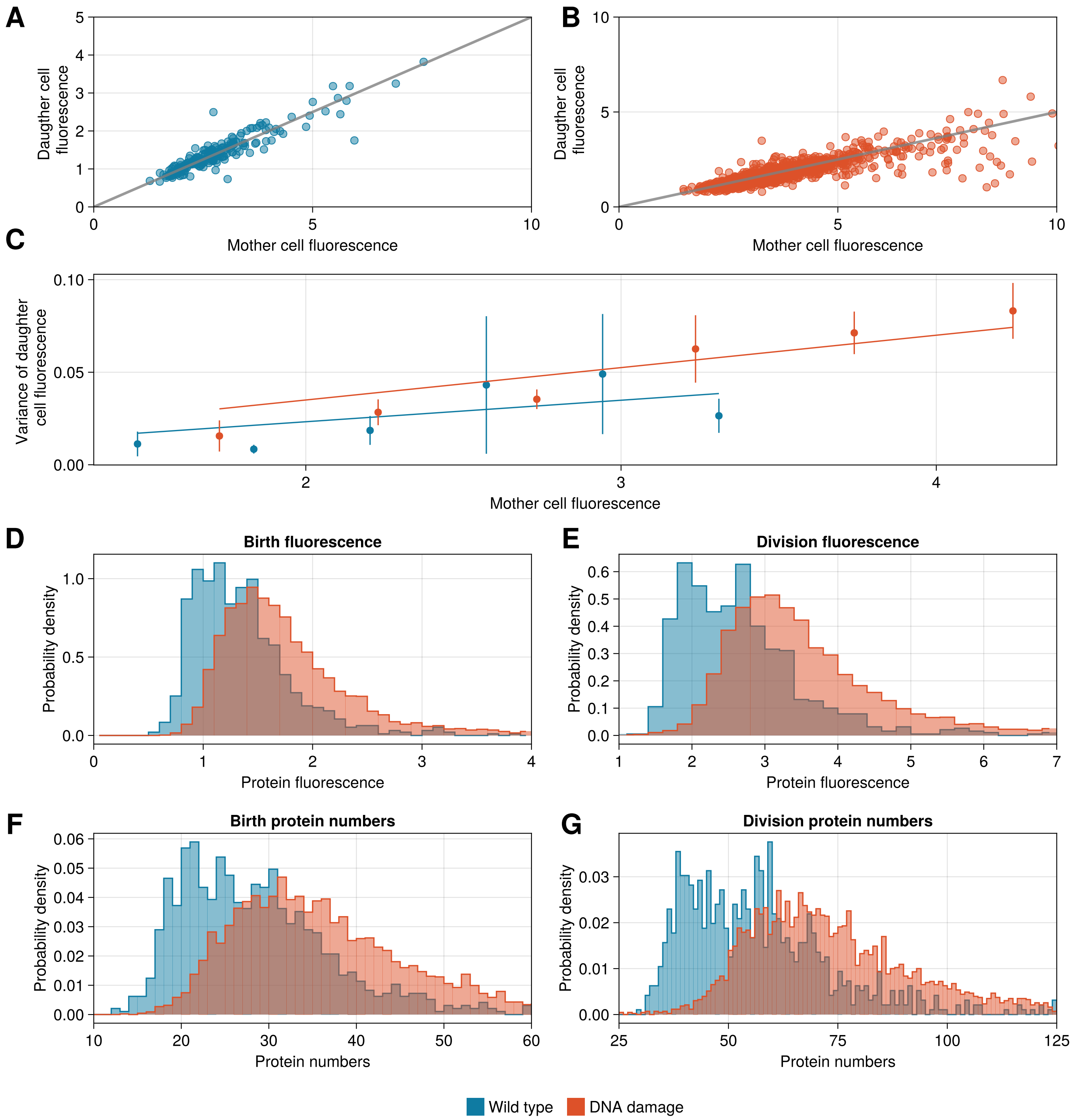

FIG. S8. Conversion factor estimation for the DNA damage response dataset. **(A-B)** The partitioning of fluorescence between the mother and daughter cells compared against symmetric partitioning (gray line with slope 0.5). **(C)** Linear regression lines for the binned mother cell fluorescence values against the variance of the daughter cell fluorescence within the bin. Scatter points represent the midpoints of the bins. The slope of the wild type case is chosen as the conversion factor. **(D-E)** Histograms displaying the birth (panel **D**) and division (panel **e**) fluorescence distributions of the wild type (blue) and the induced DNA damage strain (red) from the data. **(F-G)** Histograms showing the birth (panel **F**) and division (panel **G**) protein distribution resulting from the estimated conversion factor.

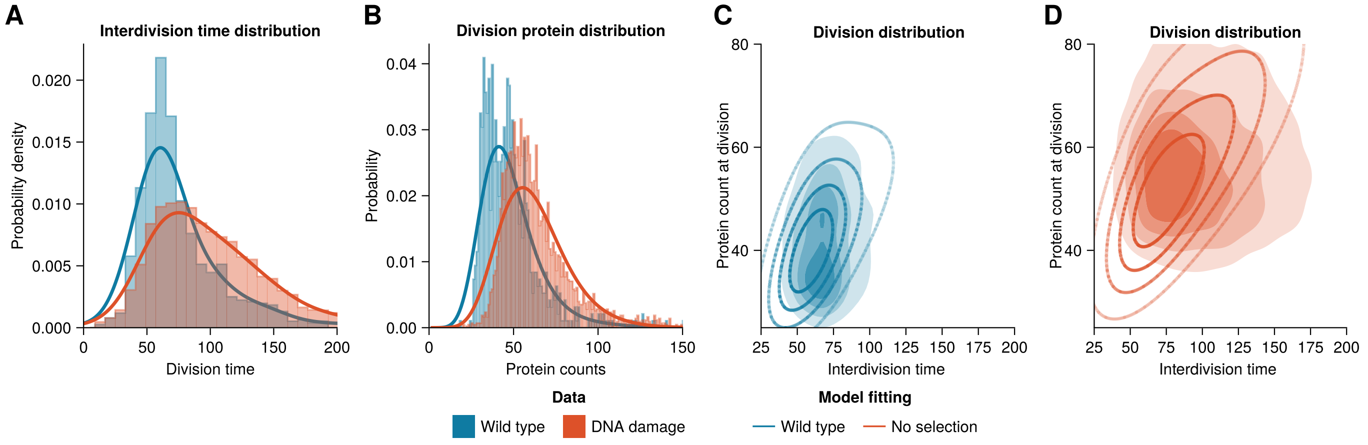

FIG. S9. Changes in the division rate without selection effects do not capture the SOS response dynamics observed in the data. **(A)** Interdivision time distributions for the two conditions are fitted by a separate kernel density estimate. **(B)** With the gene expression dynamics kept constant between the conditions the division protein distribution is well captured by the model. **(C-D)** Comparison of the division distributions of the wild type (panel **C**) and damage-induced (panel **D**) cells from the data with the mother machine lineage division distribution. The model overestimates the correlation between the protein count and interdivision time for the damage-induced cells.

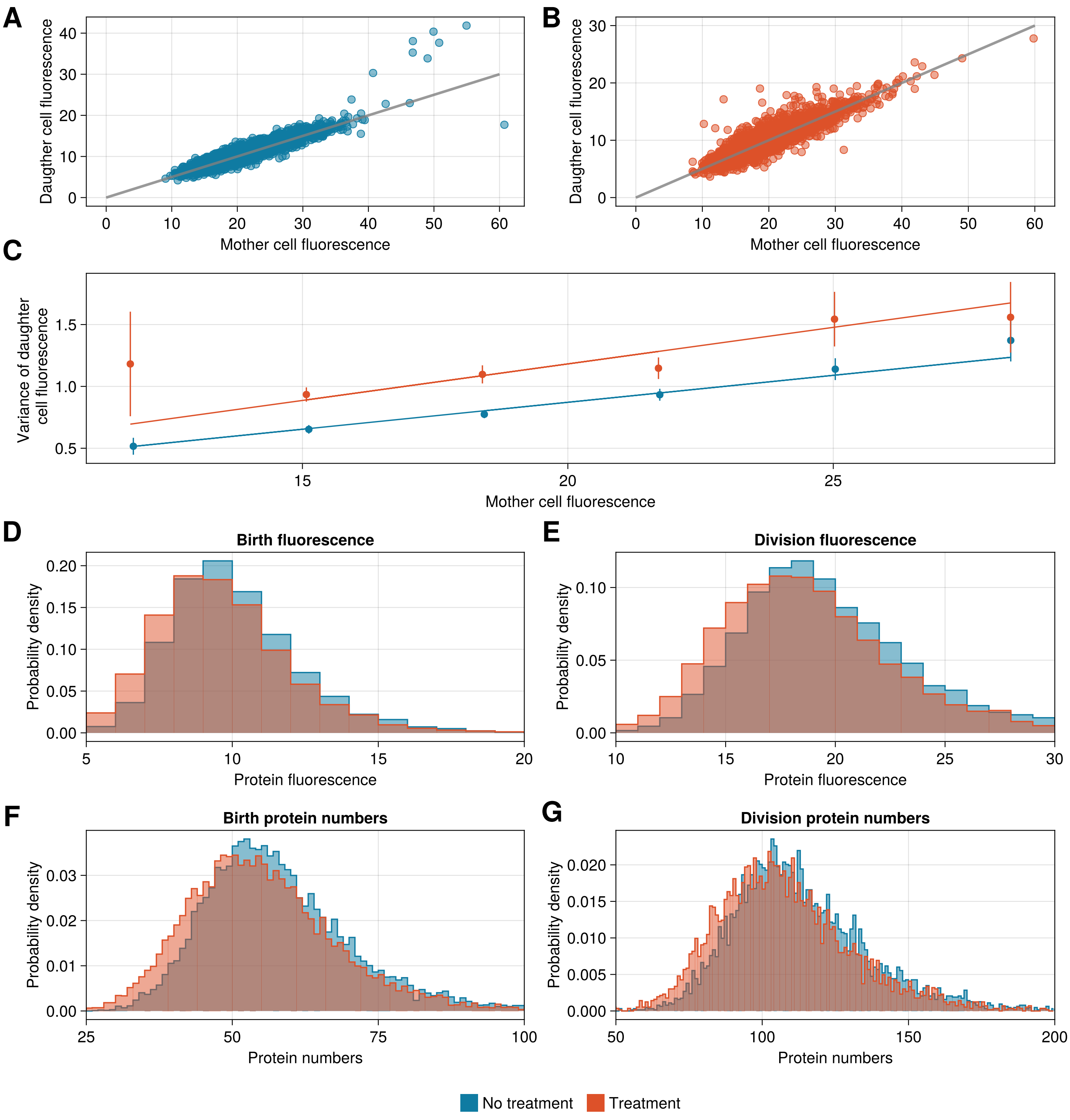

FIG. S10. Conversion factor estimation for the DNA damage response dataset. **(A-B)** The partitioning of fluorescence between the mother and daughter cells compared against symmetric partitioning (gray line with slope 0.5). **(C)** Linear regression lines for the binned mother cell fluorescence values against the variance of the daughter cell fluorescence within the bin. Scatter points represent the midpoints of the bins. The slope of the no treatment case is chosen as the conversion factor. **(D-E)** Histograms displaying the birth (panel **D**) and division (panel **E**) fluorescence distributions of the untreated (blue) and with antibiotic-treated (red) cells from the data. **(F-G)** Histograms showing the birth (panel **F**) and division (panel **G**) protein distribution resulting from the estimated conversion factor.

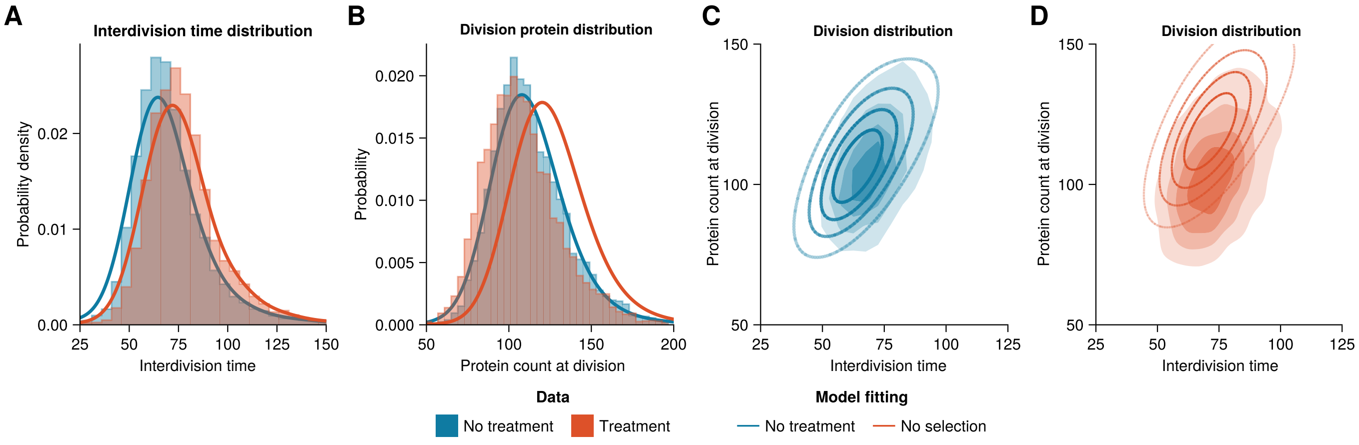

FIG. S11. Changes in the division rate alone are not sufficient to capture the antibiotic treatment response of *Escherichia coli* cells. **(A)** Interdivision time distributions of for conditions are fitted by a separate kernel density estimate. **(B)** With the gene expression dynamics kept constant between the conditions the model predicts significantly higher division protein counts than observed in the antibiotic-treated data. **(C-D)** Comparison of the division distributions of untreated (panel **C**) and antibiotic-treated (panel **D**) cells from the data with the corresponding agent-based lineage tree division distributions.

REFERENCES AND NOTES

1. H. H. McAdams, A. Arkin, Stochastic mechanisms in gene expression. *Proc. Natl. Acad. Sci. U.S.A.* **94**, 814–819 (1997).

2. M. B. Elowitz, A. J. Levine, E. D. Siggia, P. S. Swain, Stochastic gene expression in a single cell. *Science* **297**, 1183–1186 (2002).

3. J. M. Raser, E. K. O'shea, Noise in gene expression: Origins, consequences, and control. *Science* **309**, 2010–2013 (2005).

4. M. Ackermann, A functional perspective on phenotypic heterogeneity in microorganisms. *Nat. Rev. Microbiol.* **13**, 497–508 (2015).

5. B. M. Martins, J. C. Locke, Microbial individuality: How single-cell heterogeneity enables population level strategies. *Curr. Opin. Microbiol.* **24**, 104–112 (2015).

6. H. Y. Kueh, A. Champhekar, S. L. Nutt, M. B. Elowitz, E. V. Rothenberg, Positive feedback between PU. Positive feedback between PU.1 and the cell cycle controls myeloid differentiation. *Science* **341**, 670–673 (2013).

7. S. Furutachi, H. Miya, T. Watanabe, H. Kawai, N. Yamasaki, Y. Harada, I. Imayoshi, M. Nelson, K. I. Nakayama, Y. Hirabayashi, Y. Gotoh, Slowly dividing neural progenitors are an embryonic origin of adult neural stem cells. *Nat. Neurosci.* **18**, 657–665 (2015).

8. A. R. Barr, S. Cooper, F. S. Heldt, F. Butera, H. Stoy, J. Mansfeld, B. Novák, C. Bakal, DNA damage during S-phase mediates the proliferation-quiescence decision in the subsequent G1 via p21 expression. *Nat. Commun.* **8**, 14728 (2017).

9. O. Patange, C. Schwall, M. Jones, C. Villava, D. A. Griffith, A. Phillips, J. C. Locke, *Escherichia coli* can survive stress by noisy growth modulation. *Nat. Commun.* **9**, 5333 (2018).

10. N. M. Sampaio, C. M. Blassick, V. Andreani, J.-B. Lugagne, M. J. Dunlop, Dynamic gene expression and growth underlie cell-to-cell heterogeneity in *Escherichia coli* stress response. *Proc. Natl. Acad. Sci. U.S.A.* **119**, e2115032119 (2022).

11. N. Q. Balaban, J. Merrin, R. Chait, L. Kowalik, S. Leibler, Bacterial persistence as a phenotypic switch. *Science* **305**, 1622–1625 (2004).

12. Y. Wakamoto, N. Dhar, R. Chait, K. Schneider, F. Signorino-Gelo, S. Leibler, J. D. McKinney, Dynamic persistence of antibiotic-stressed mycobacteria. *Science* **339**, 91–95 (2013).

13. L. Gerosa, C. Chidley, F. Fröhlich, G. Sanchez, S. K. Lim, J. Muhlich, J.-Y. Chen, S. Vallabhaneni, G. J. Baker, D. Schapiro, M. I. Atanasova, L. A. Chylek, T. Shi, L. Yi, C. D. Nicora, A. Claas, T. S. C. Ng, R. H. Kohler, D. A. Lauffenburger, R. Weissleder, M. A. Miller, W. J. Qian, H. S. Wiley, P. K. Sorger, Receptor-driven ERK pulses reconfigure MAPK signaling and enable persistence of drug-adapted BRAF-mutant melanoma cells. *Cell Syst.* **11**, 478–494.e9 (2020).

14. C. Tan, P. Marguet, L. You, Emergent bistability by a growth-modulating positive feedback circuit. *Nat. Chem. Biol.* **5**, 842–848 (2009).

15. A. Y. Weiße, D. A. Oyarzún, V. Danos, P. S. Swain, Mechanistic links between cellular trade-offs, gene expression, and growth. *Proc. Natl. Acad. Sci. U.S.A.* **112**, E1038–E1047 (2015).

16. C. Aditya, F. Bertaux, G. Batt, J. Ruess, Using single-cell models to predict the functionality of synthetic circuits at the population scale. *Proc. Natl. Acad. Sci.* **119**, e2114438119 (2022).

17. P. S. Swain, M. B. Elowitz, E. D. Siggia, Intrinsic and extrinsic contributions to stochasticity in gene expression. *Proc. Natl. Acad. Sci. U.S.A.* **99**, 12795–12800 (2002).

18. D. Volfson, J. Marciniak, W. J. Blake, N. Ostroff, L. S. Tsimring, J. Hasty, Origins of extrinsic variability in eukaryotic gene expression. *Nature* **439**, 861–864 (2006).

19. D. Huh, J. Paulsson, Non-genetic heterogeneity from stochastic partitioning at cell division. *Nat. Genet.* **43**, 95–100 (2011).

20. Z. Cao, R. Grima, Analytical distributions for detailed models of stochastic gene expression in eukaryotic cells. *Proc. Natl. Acad. Sci. U.S.A.* **117**, 4682–4692 (2020).

21. M. Hashimoto, T. Nozoe, H. Nakaoka, R. Okura, S. Akiyoshi, K. Kaneko, E. Kussell, Y. Wakamoto, Noise-driven growth rate gain in clonal cellular populations. *Proc. Natl. Acad. Sci. U.S.A.* **113**, 3251–3256 (2016).

22. P. Thomas, Making sense of snapshot data: Ergodic principle for clonal cell populations. *J. R. Soc. Interface* **14**, 20170467 (2017).

23. P. Thomas, Intrinsic and extrinsic noise of gene expression in lineage trees. *Sci. Rep.* **9**, 474 (2019).

24. D. J. Kiviet, P. Nghe, N. Walker, S. Boulineau, V. Sunderlikova, S. J. Tans, Stochasticity of metabolism and growth at the single-cell level. *Nature* **514**, 376–379 (2014).

25. P. Thomas, G. Terradot, V. Danos, A. Y. Weiße, Sources, propagation and consequences of stochasticity in cellular growth. *Nat. Commun.* **9**, 4528 (2018).

26. F. A. Hughes, A. R. Barr, P. Thomas, Patterns of interdivision time correlations reveal hidden cell cycle factors. *eLife* **11**, e80927 (2022).

27. K. R. Ghusinga, C. A. Vargas-Garcia, A. Singh, A mechanistic stochastic framework for regulating bacterial cell division. *Sci. Rep.* **6**, 30229 (2016).

28. J. Kuntz, P. Thomas, G.-B. Stan, M. Barahona, The exit time finite state projection scheme: Bounding exit distributions and occupation measures of continuous-time Markov chains. *SIAM J. Sci. Comput.* **41**, A748–A769 (2019).

29. M. Panlilio, J. Grilli, G. Tallarico, I. Iuliani, B. Sclavi, P. Cicuta, M. Cosentino Lagomarsino, Threshold accumulation of a constitutive protein explains E. coli cell-division behavior in nutrient upshifts. *Proc. Natl. Acad. Sci. U.S.A.* **118**, e2016391118 (2021).

30. K. Biswas, N. Brenner, Universality of phenotypic distributions in bacteria. bioRxiv [Preprint]. 2024. https://doi.org/10.1101/2022.08.21.504683.

31. L. Luo, Y. Bai, X. Fu, Stochastic threshold in cell size control. *Phys. Rev. Res.* **5**, 013173 (2023).

32. T. Mora, A. M. Walczak, Effect of phenotypic selection on stochastic gene expression. *J. Phys. Chem. B* **117**, 13194–13205 (2013).

33. M. Ciechonska, M. Sturrock, A. Grob, G. Larrouy-Maumus, V. Shahrezaei, M. Isalan, Emergent expression of fitness-conferring genes by phenotypic selection. *PNAS nexus* **1**, pgac069 (2022).

34. P. Wang, L. Robert, J. Pelletier, W. L. Dang, F. Taddei, A. Wright, S. Jun, Robust growth of *Escherichia coli*. *Curr. Biol.* **20**, 1099–1103 (2010).

35. H. Von Foerster, in *The Kinetics of Cellular Proliferation*, F. Stohlman, ed. (Grune and Stratton, 1959), pp. 382–407.

36. T. Nozoe, E. Kussell, Y. Wakamoto, Inferring fitness landscapes and selection on phenotypic states from single-cell genealogical data. *PLOS Genet.* **13**, e1006653 (2017).

37. S. Yamauchi, T. Nozoe, R. Okura, E. Kussell, Y. Wakamoto, A unified framework for measuring selection on cellular lineages and traits. *eLife* **11**, e72299 (2022).

38. Y. Sughiyama, T. J. Kobayashi, K. Tsumura, K. Aihara, Pathwise thermodynamic structure in population dynamics. *Phys. Rev. E* **91**, 032120 (2015).

39. A. Genthon, D. Lacoste, Universal constraints on selection strength in lineage trees. *Phys. Rev. Res.* **3**, 023187 (2021).

40. O. G. Berg, A model for the statistical fluctuations of protein numbers in a microbial population. *J. Theor. Biol.* **71**, 587–603 (1978).

41. I. G. Johnston, N. S. Jones, Closed-form stochastic solutions for non-equilibrium dynamics and inheritance of cellular components over many cell divisions. *Proc. R. Soc. A Math. Phys. Eng. Sci.* **471**, 20150050 (2015).

42. B. Munsky, M. Khammash, The finite state projection algorithm for the solution of the chemical master equation. *J. Chem. Phys.* **124**, 044104 (2006).

43. E. O. Powell, Growth rate and generation time of bacteria, with special reference to continuous culture. *Microbiology* **15**, 492–511 (1956).

44. P. Thomas, N. Popović, R. Grima, Phenotypic switching in gene regulatory networks. *Proc. Natl. Acad. Sci. U.S.A.* **111**, 6994–6999 (2014).

45. P. J. Choi, L. Cai, K. Frieda, X. S. Xie, A stochastic single-molecule event triggers phenotype switching of a bacterial cell. *Science* **322**, 442–446 (2008).

46. M. A. Coomer, L. Ham, M. P. Stumpf, Noise distorts the epigenetic landscape and shapes cell-fate decisions. *Cell Syst.* **13**, 83–102.e6 (2022).

47. S. Jaramillo-Riveri, J. Broughton, A. McVey, T. Pilizota, M. Scott, M. El Karoui, Growth-dependent heterogeneity in the DNA damage response in *Escherichia coli*. *Mol. Syst. Biol.* **18**, e10441 (2022).

48. V. Shahrezaei, P. S. Swain, Analytical distributions for stochastic gene expression. *Proc. Natl. Acad. Sci. U.S.A.* **105**, 17256–17261 (2008).

49. B. Shahriari, K. Swersky, Z. Wang, R. P. Adams, N. de Freitas, Taking the human out of the loop: A review of bayesian optimization. *Proc. IEEE* **104**, 148–175 (2016).

50. J.-W. Veening, W. K. Smits, O. P. Kuipers, Bistability, epigenetics, and bet-hedging in bacteria. *Annu. Rev. Microbiol.* **62**, 193–210 (2008).

51. A. C. S. Jørgensen, A. Ghosh, M. Sturrock, V. Shahrezaei, Efficient Bayesian inference for stochastic agent-based models. *PLoS Comput. Biol.* **18**, e1009508 (2022).

52. I. G. Ion, C. Wildner, D. Loukrezis, H. Koeppl, H. De Gersem, Tensor-train approximation of the chemical master equation and its application for parameter inference. *J. Chem. Phys.* **155**, 034102 (2021).

53. L. Liu, W. Michowski, A. Kolodziejczyk, P. Sicinski, The cell cycle in stem cell proliferation, pluripotency and differentiation. *Nat. Cell Biol.* **21**, 1060–1067 (2019).

54. E. Fuchs, The tortoise and the hair: Slow-cycling cells in the stem cell race. *Cell* **137**, 811–819 (2009).

55. J. Lin, A. Amir, Homeostasis of protein and mRNA concentrations in growing cells. *Nat. Commun.* **9**, 4496 (2018).

56. B. M. C. Martins, A. K. Tooke, P. Thomas, J. C. W. Locke, Cell size control driven by the circadian clock and environment in cyanobacteria. *Proc. Natl. Acad. Sci.* **115**, E11415–E11424 (2018).

57. I. G. Johnston, B. Gaal, R. P. des Neves, T. Enver, F. J. Iborra, N. S. Jones, Mitochondrial variability as a source of extrinsic cellular noise. *PLoS Comput. Biol.* **8**, e1002416 (2012).

58. D. Shiokawa, H. Sakai, H. Ohata, T. Miyazaki, Y. Kanda, S. Sekine, D. Narushima, M. Hosokawa, M. Kato, Y. Suzuki, H. Takeyama, H. Kambara, H. Nakagama, K. Okamoto, Slow-cycling cancer stem cells regulate progression and chemoresistance in colon cancer. *Cancer Res.* **80**, 4451–4464 (2020).

59. M. Voliotis, P. Thomas, R. Grima, C. G. Bowsher, Stochastic simulation of biomolecular networks in dynamic environments. *PLoS Comput. Biol.* **12**, e1004923 (2016).

60. P. Thomas, V. Shahrezaei, Coordination of gene expression noise with cell size: Analytical results for agent-based models of growing cell populations. *J. R. Soc. Interface* **18**, 20210274 (2021).

61. O. Diekmann, H. A. Lauwerier, T. Aldenberg, J. A. J. Metz, Growth, fission and the stable size distribution. *J. Math. Biol.* **18**, 135–148 (1983).

62. FiniteStateProjection.jl, https://github.com/SciML/FiniteStateProjection.jl [accessed 15 February 2024].

63. T. E. Loman, Y. Ma, V. Ilin, S. Gowda, N. Korsbo, N. Yewale, C. Rackauckas, S. A. Isaacson, Catalyst: Fast and flexible modeling of reaction networks. *PLoS Comput. Biol.* **19**, e1011530 (2023).

64. C. Gardiner, *Stochastic Methods: A Handbook for the Natural and Social Sciences*, Springer Series in Synergetics (Springer, 2009).

65. N. Rosenfeld, J. W. Young, U. Alon, P. S. Swain, M. B. Elowitz, Gene regulation at the single-cell level. *Science* **307**, 1962–1965 (2005).

66. scikit-optimize: Sequential model-based optimization in Python, https://scikit-optimize.github.io/dev/ [accessed 7 February 2024].
